# Supplementary material for: Optimal shrinkage denoising breaks the noise floor in high-resolution diffusion MRI
Source: Patterns (N Y). 2024 Mar 14;5(4):100954. doi: 10.1016/j.patter.2024.100954 (PMC11026978; doi:10.1016/j.patter.2024.100954)
Supplement: Document S1. Figures S1–S7 and Tables S1 and S2 [file mmc1.pdf]

**Patterns, Volume *5***

# Supplemental information

# Optimal shrinkage denoising breaks the noise floor in high-resolution diffusion MRI

**Khoi Huynh, Wei-Tang Chang, Ye Wu, and Pew-Thian Yap**

# Supplementary Materials

Fig. S1. **Data simulation.** Phantom (a) used to simulate noise-free ground truth images (b). The white arrows mark CSF-like regions with isotropic diffusion. Background phase (c), spatially-varying noise map (d), and exemplar channel-sensitivity maps (e, only 4 shown here) used with Eq. (23) to synthesize a realistic dataset.

Fig. S2. **Free-water diffusion.** Normalized free-water diffusion signal from ground truth, noisy, and denoised data with 26 volumes. Shaded regions indicate the original noise floor. The zoomed-in plots highlight the differences between MCC methods.

Fig. S3. **Free-water diffusion.** Normalized free-water diffusion signal from ground truth, noisy, and denoised data with 47 volumes. Shaded regions indicate the original noise floor. The zoomed-in plots highlight the differences between MCC methods.

Fig. S4. **Free-water diffusion.** Normalized free-water diffusion signal from ground truth, noisy, and denoised data with 89 volumes. Shaded regions indicate the original noise floor. The zoomed-in plots highlight the differences between MCC methods.

Fig. S5. **Noise mapping.** Noise map estimated from different approaches. The approach used in our framework (right most) is closest to the ground truth (GT).

Fig. S6. **Background phase estimation.** Example background phases estimated during phase unwinding in different MCC denoising strategies. Also shown are the background phases for noisy data and the ground truth (for simulated data only).

Fig. S7. **MRtrix vs Dipy MP-PCA.** The absolute relative difference between MRtrix *dwidenoise* and Dipy *dipy_denoise_mppca* is less than 1%.

Table S1. **Tractometer statistics for synthetic data.**

Table S2. **Summary of methods evaluated.**

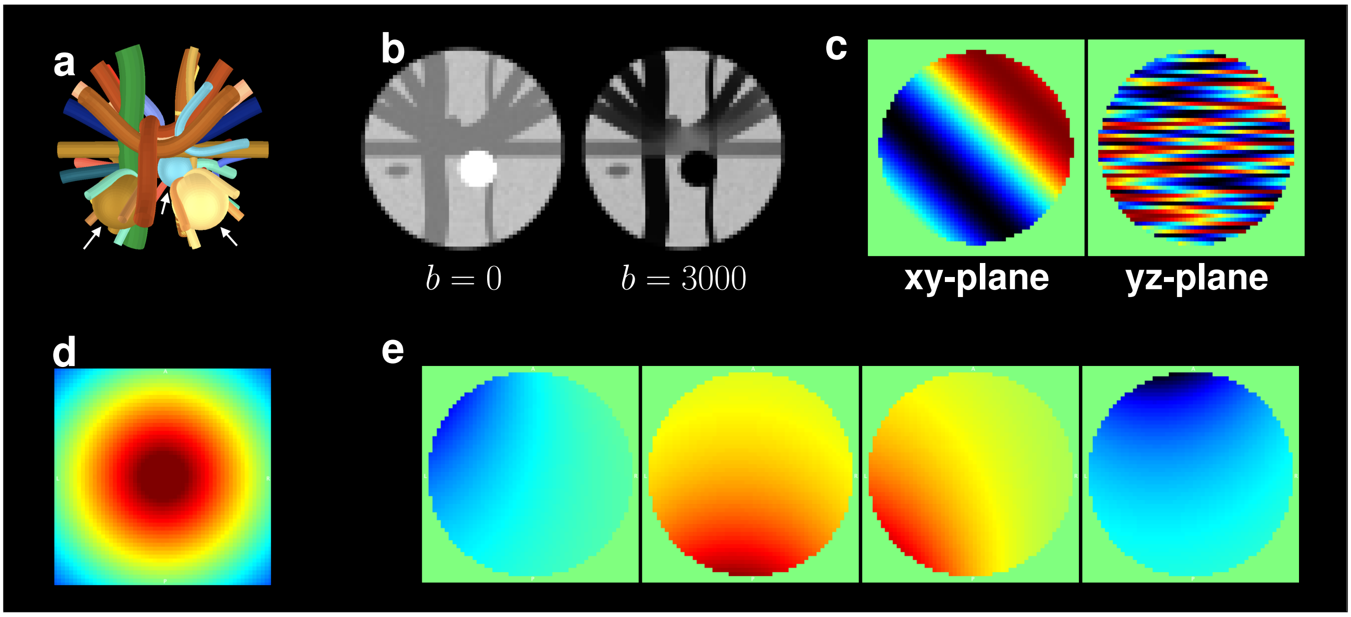


Figure S1: **Data simulation.** Phantom (a) used to simulate noise-free ground truth images (b). The white arrows mark CSF-like regions with isotropic diffusion. Background phase (c), spatially-varying noise map (d), and exemplar channel-sensitivity maps (e, only 4 shown here) used with Eq. (23) to synthesize a realistic dataset.

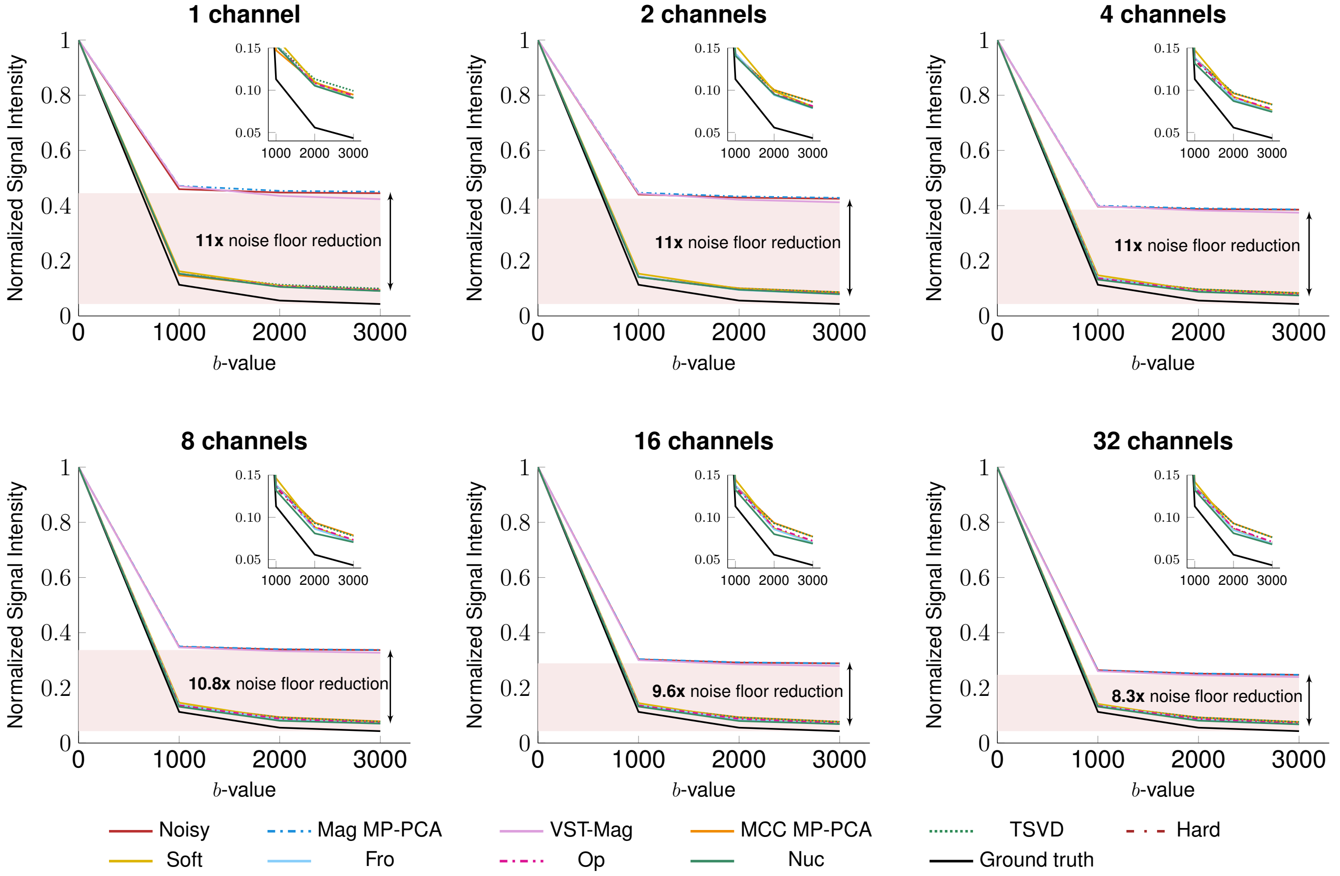


Figure S2: **Free-water diffusion.** Normalized free-water diffusion signal from ground truth, noisy, and denoised data with 26 volumes. Shaded regions indicate the original noise floor. The zoomed-in plots highlight the differences between MCC methods.

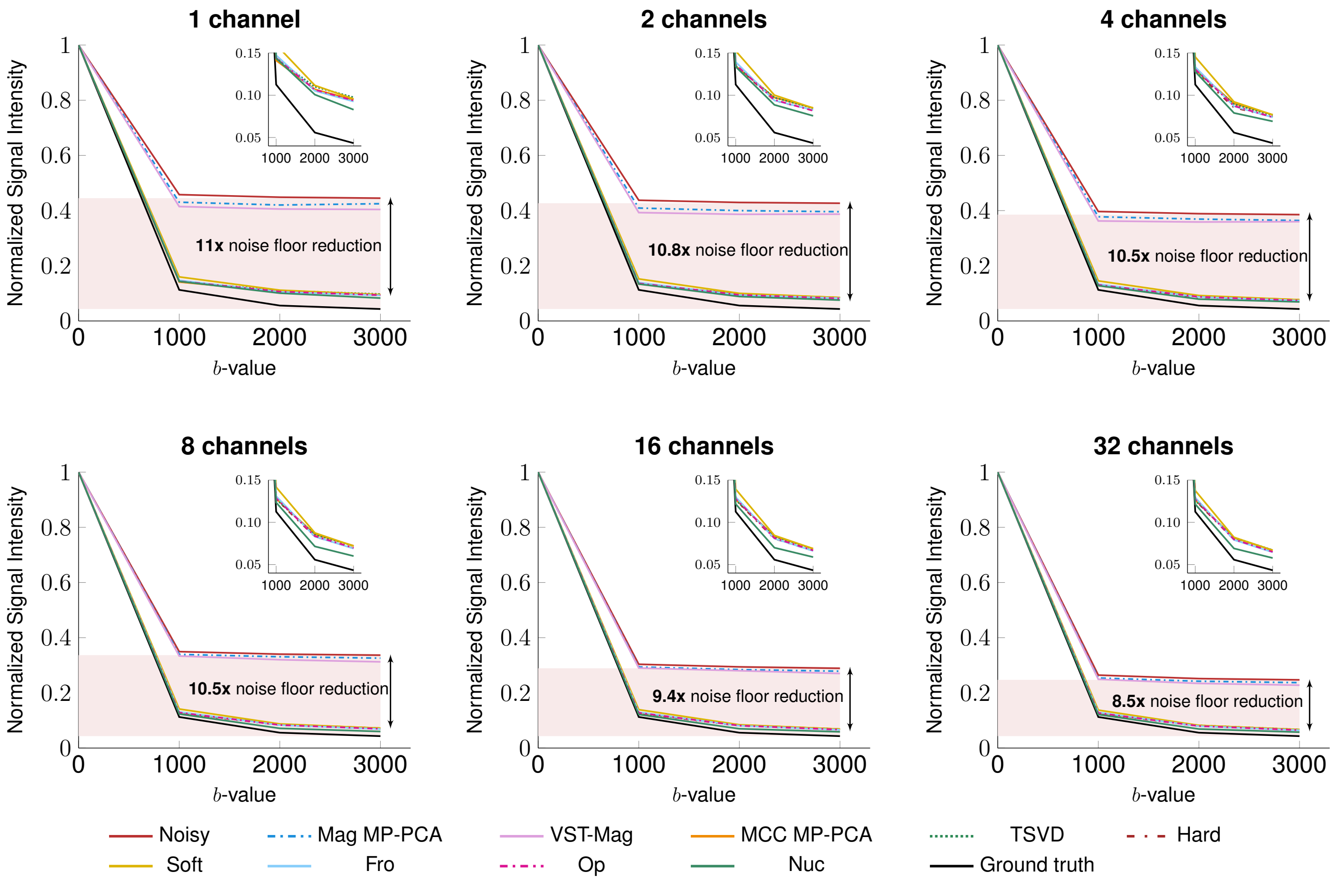


Figure S3: **Free-water diffusion.** Normalized free-water diffusion signal from ground truth, noisy, and denoised data with 47 volumes. Shaded regions indicate the original noise floor. The zoomed-in plots highlight the differences between MCC methods.

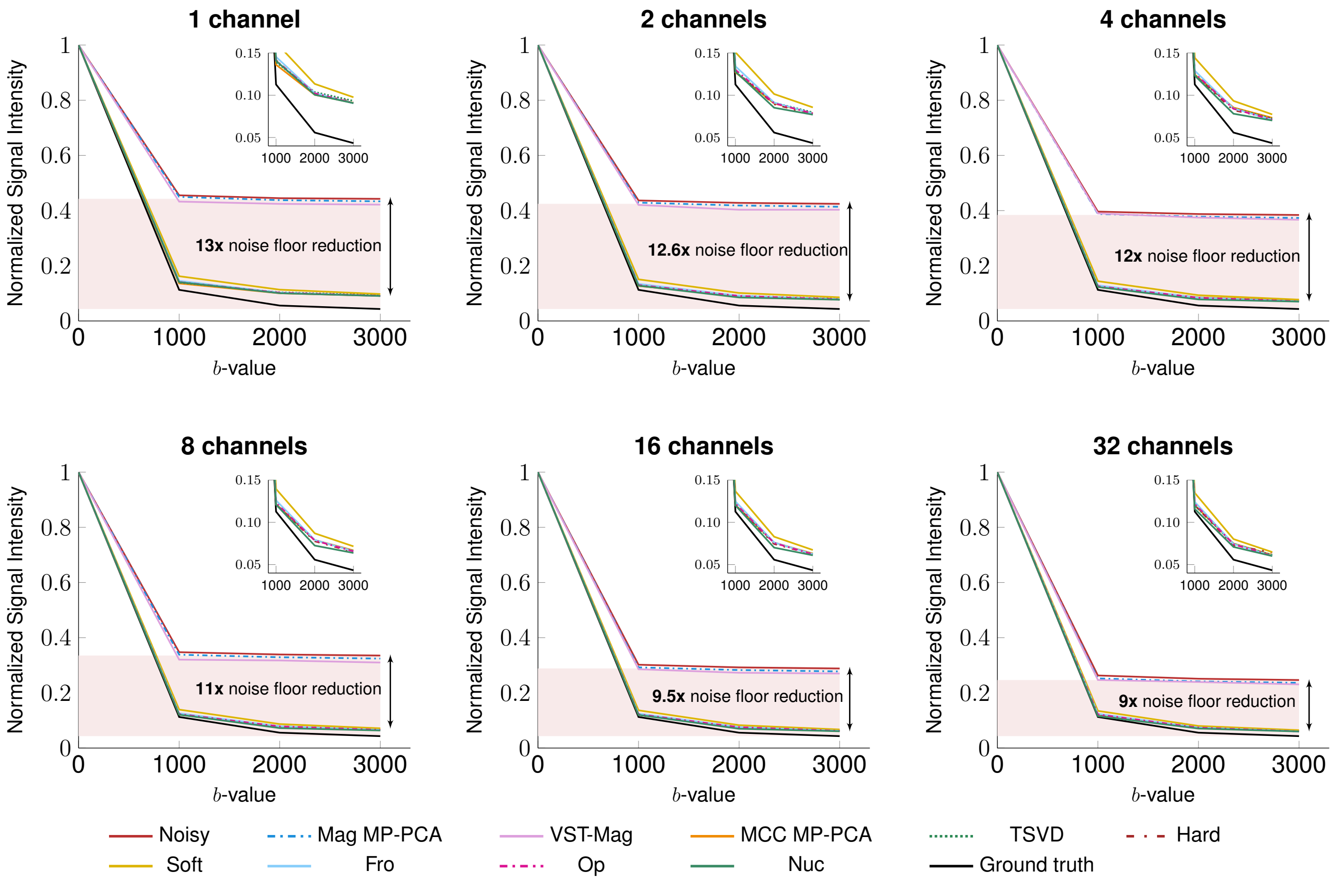


Figure S4: **Free-water diffusion.** Normalized free-water diffusion signal from ground truth, noisy, and denoised data with 89 volumes. Shaded regions indicate the original noise floor. The zoomed-in plots highlight the differences between MCC methods.

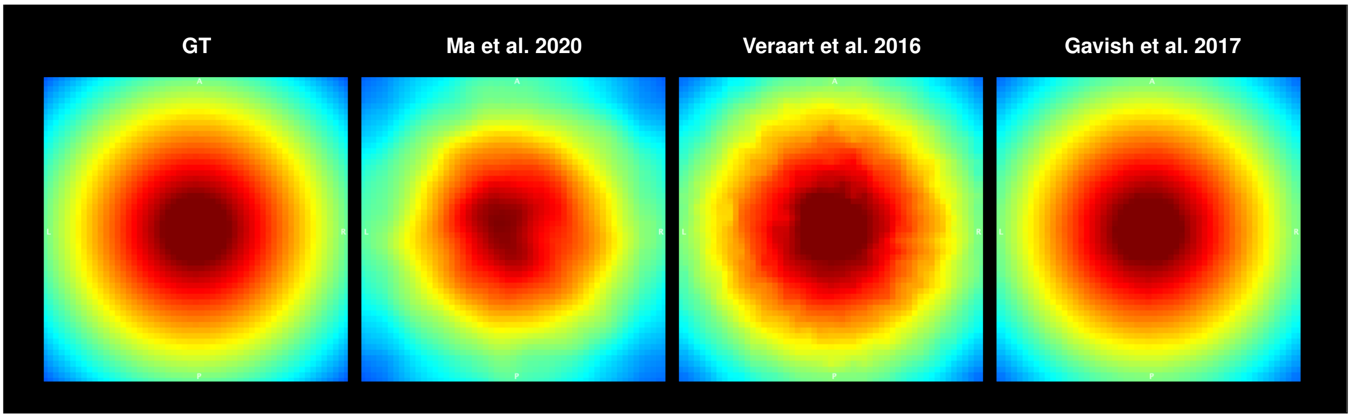


Figure S5: **Noise mapping.** Noise map estimated from different approaches. The approach used in our framework (right most) is closest to the ground truth (GT).

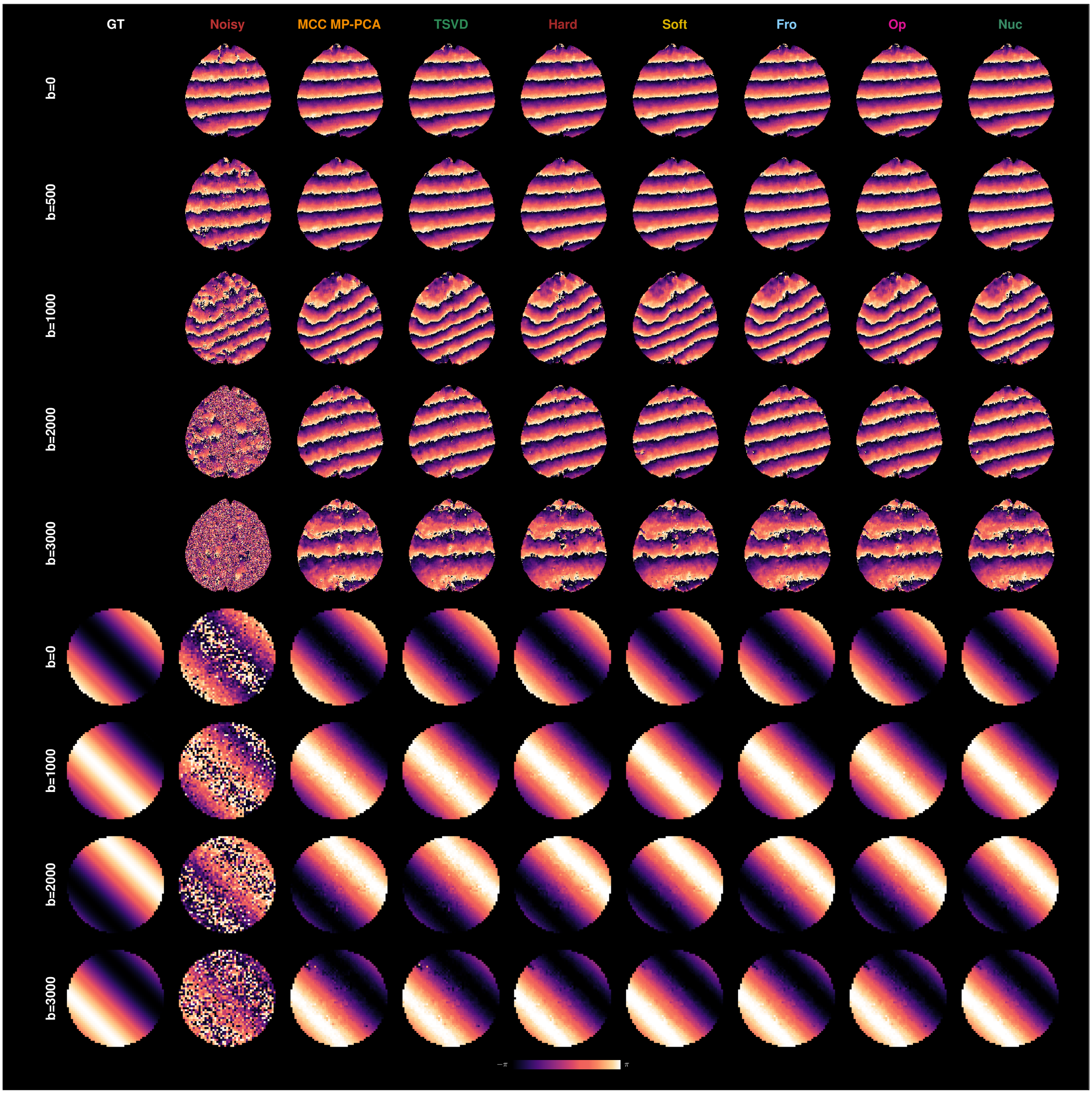


Figure S6: **Background phase estimation.** Example background phases estimated during phase unwinding in different MCC denoising strategies. Also shown are the background phases for noisy data and the ground truth (for simulated data only).

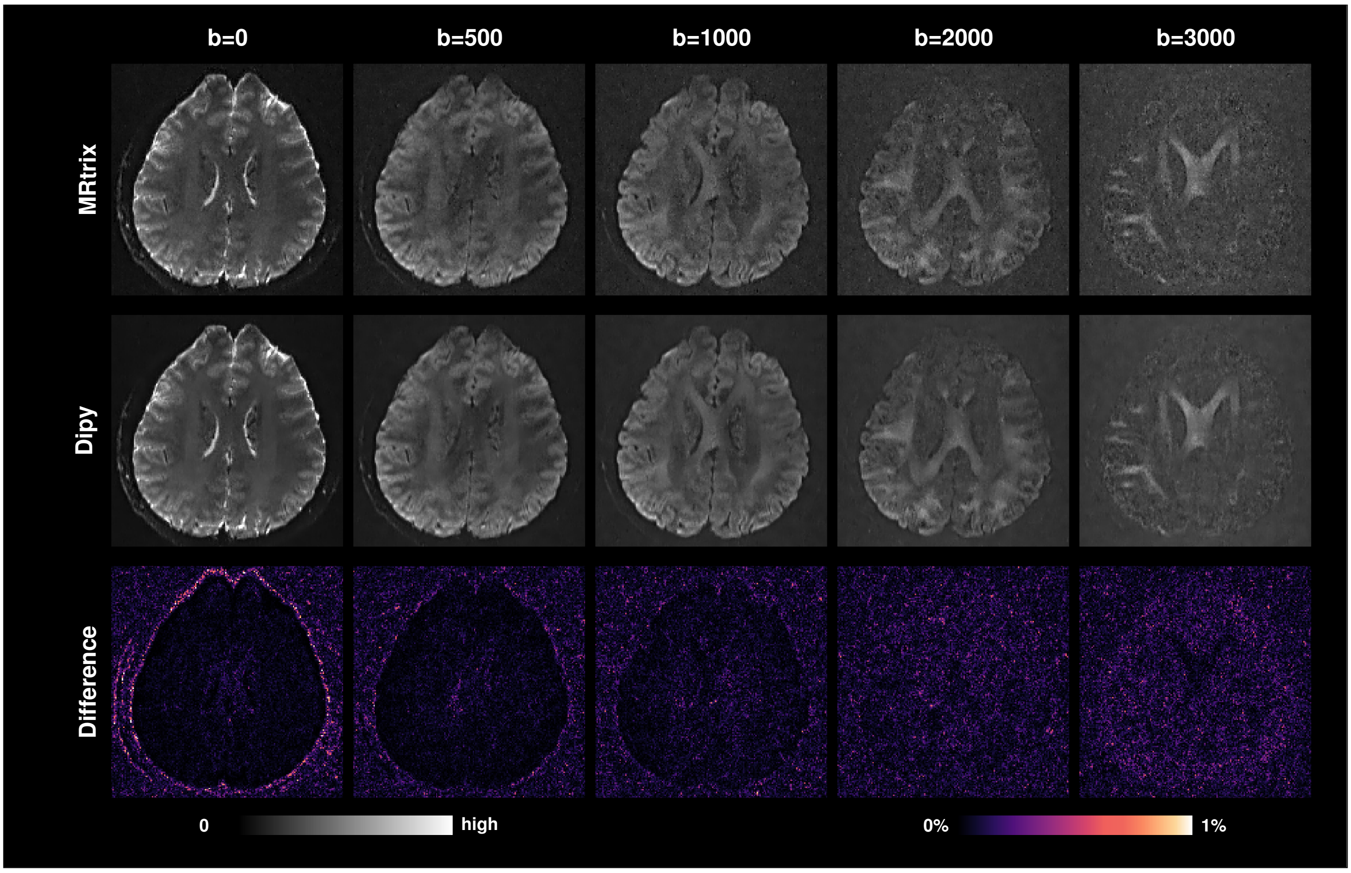


Figure S7: **MRtrix vs Dipy MP-PCA.** The absolute relative difference between MRtrix *dwidenoise* and Dipy *dipy_denoise_mppca* is less than 1%.

Table S1: Tractometer statistics for synthetic data.

| | VC(%) | IC(%) | NC(%) | Score | VB | IB |
|---|---|---|---|---|---|---|
| **Ground Truth** | 41.00 | 25.64 | 33.37 | 0.48 | 26 | 1 |
| **Noisy** | 2.17 | 5.01 | 92.82 | 0.04 | 17 | 2 |
| **Mag MP-PCA** | 16.03 | 10.13 | 73.85 | 0.21 | 16 | 2 |
| **VST-Mag** | 20.19 | 14.15 | 65.66 | 0.26 | 15 | 1 |
| **MCC MP-PCA** | 36.75 | 18.94 | 44.31 | 0.43 | 22 | 1 |
| **TSVD** | 39.58 | 19.63 | 40.79 | 0.46 | 23 | 1 |
| **Hard** | 37.96 | 18.07 | 43.96 | 0.44 | 21 | 1 |
| **Soft** | 35.57 | 19.80 | 44.63 | 0.43 | 21 | 1 |
| **Fro** | 38.03 | 19.15 | 42.82 | 0.45 | 23 | 1 |
| **Op** | 39.45 | 19.32 | 41.23 | 0.46 | 24 | 1 |
| **Nuc** | 39.83 | 19.67 | 40.50 | 0.47 | 25 | 1 |

Table S2: Summary of methods evaluated.

| | Value | Channel | Channel Decorrelation | Phase Unwinding | Recovery Shrinkage | Noise Estimation |
|---|---|---|---|---|---|---|
| **Mag MP-PCA**[11] | Magnitude | Single | N/A | N/A | Eq. (4) | Eq. (15) |
| **VST-Mag**[8] | Magnitude | Single | N/A | N/A | Eq. (7) | Two steps, see[8] |
| **MCC MP-PCA** | Complex | Multi | Yes | Yes | Eq. (4) | Eq. (15) |
| **TSVD** | Complex | Multi | Yes | Yes | Eq. (11) | Eq. (16) |
| **Hard** | Complex | Multi | Yes | Yes | Eq. (12) | Eq. (16) |
| **Soft** | Complex | Multi | Yes | Yes | Eq. (14) | Eq. (16) |
| **Fro** | Complex | Multi | Yes | Yes | Eq. (7) | Eq. (16) |
| **Op** | Complex | Multi | Yes | Yes | Eq. (9) | Eq. (16) |
| **Nuc** | Complex | Multi | Yes | Yes | Eq. (8) | Eq. (16) |
