# Supplementary material for: Optimal shrinkage denoising breaks the noise floor in high-resolution diffusion MRI
Source: Patterns (N Y). 2024 Mar 14;5(4):100954. doi: 10.1016/j.patter.2024.100954 (PMC11026978; doi:10.1016/j.patter.2024.100954)
Supplement: Document S2. Article plus supplemental information [file mmc2.pdf]

Patterns

Article

Optimal shrinkage denoising breaks the noise floor in high-resolution diffusion MRI

Graphical abstract

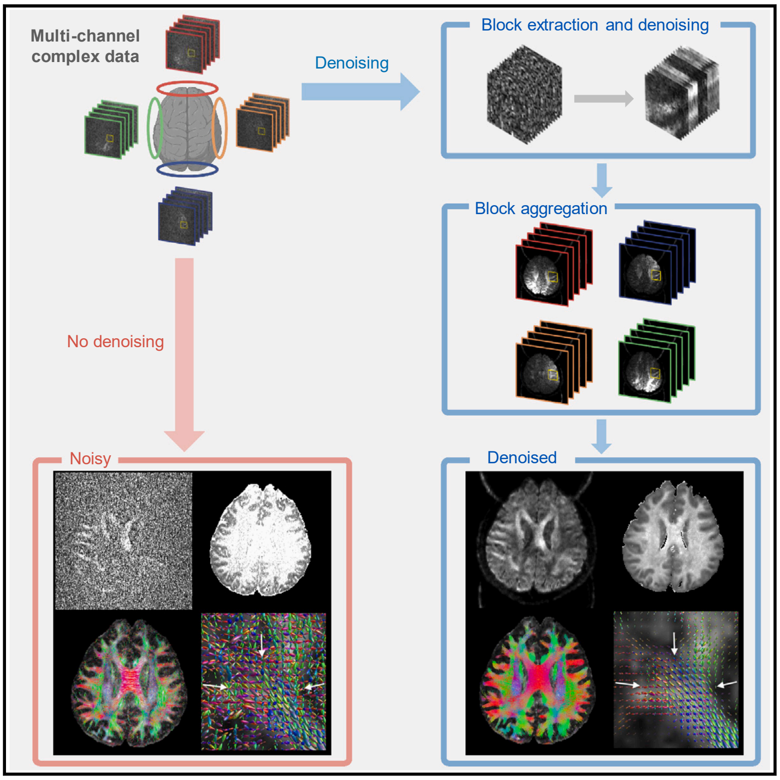

Highlights

- MRI resolution is bounded by the level of thermal noise
- Mitigating noise typically requires time-consuming scans or expensive hardware upgrades
- We leverage redundancy in multi-channel-complex-valued data to reduce noise
- Noise reduction improves downstream analyses

Authors

Khoi Huynh, Wei-Tang Chang, Ye Wu, Pew-Thian Yap

Correspondence

ptyap@med.unc.edu

In brief

Detailed MRI of soft tissues is often marred by noise. This study presents a strategy to substantially reduce noise by leveraging the inherent redundancy of MRI data, eliminating the need for lengthy scans and costly hardware upgrades. Noise reduction greatly enhances the characterization of tissue microstructure and white matter pathways.

Huynh et al., 2024, Patterns *5*, 100954
April 12, 2024 © 2024 The Author(s).
https://doi.org/10.1016/j.patter.2024.100954

CellPress

Patterns

CellPress
OPEN ACCESS

Article

Optimal shrinkage denoising breaks the noise floor in high-resolution diffusion MRI

Khoi Huynh,[1,2] Wei-Tang Chang,[1,2] Ye Wu,[1,2] and Pew-Thian Yap[1,2,3,*]
[1]Department of Radiology, University of North Carolina at Chapel Hill, Chapel Hill, NC 27599, USA
[2]Biomedical Research Imaging Center, University of North Carolina at Chapel Hill, Chapel Hill, NC 27599, USA
[3]Lead contact
*Correspondence: ptyap@med.unc.edu
https://doi.org/10.1016/j.patter.2024.100954

THE BIGGER PICTURE Diffusion magnetic resonance imaging (MRI) is a pivotal tool in pathology research and for the study of brain development. However, noise often undermines the intricate details expected to be gained from increasing the spatial resolution. Rather than resorting to time-consuming scans and costly hardware upgrades to combat noise, data redundancy in MRI can be leveraged to effectively reduce noise, unveil details, and enhance downstream analyses.

SUMMARY

The spatial resolution attainable in diffusion magnetic resonance (MR) imaging is inherently limited by noise. The weaker signal associated with a smaller voxel size, especially at a high level of diffusion sensitization, is often buried under the noise floor owing to the non-Gaussian nature of the MR magnitude signal. Here, we show how the noise floor can be suppressed remarkably via optimal shrinkage of singular values associated with noise in complex-valued k-space data from multiple receiver channels. We explore and compare different low-rank signal matrix recovery strategies to utilize the inherently redundant information from multiple channels. In combination with background phase removal, the optimal strategy reduces the noise floor by 11 times. Our framework enables imaging with substantially improved resolution for precise characterization of tissue microstructure and white matter pathways without relying on expensive hardware upgrades and time-consuming acquisition repetitions, outperforming other related denoising methods.

INTRODUCTION

Diffusion magnetic resonance imaging (dMRI) is a unique non-invasive technique for probing brain microstructure and white matter pathways, capable of super-resolution unrestricted by the radiofrequency (RF) wavelength. However, in reality, the resolution is capped by the signal-to-noise ratio (SNR), which is proportional to voxel size. A 2-fold reduction in voxel size in each dimension is associated with an 8-fold ($2^3 = 8$) decrease in SNR. The problem is further compounded by the fact that a low-SNR magnitude signal may dip below the Rician noise floor and become unmeasurable.[1] This is a particularly severe problem for dMRI owing to the pronounced thermal noise and the low signal amplitude resulting from fast echo-planar acquisition strategies.

SNR can be enhanced with higher magnetic field strengths or better RF coils.[2] However, hardware advancement has reached its limit,[3] and ultra-high-field scanners are not yet widely available.[3,4] While SNR can alternatively be enhanced by repeating and averaging acquisitions, SNR improves slowly with the square root of the number of repetitions.[5] For example, $8^2 = 64$ repetitions are needed to compensate for an 8-fold SNR decrease. Enhancing SNR by improving hardware or repeating acquisitions is expensive, impractical, time consuming, uncomfortable for patients, and prone to motion and physiological artifacts.

Post-acquisition denoising increases SNR without requiring hardware upgrades and scan repetitions. A state-of-the-art denoising approach is based on random matrix theory (RMT).[6] This approach works akin to local principal-component analysis (PCA) denoising[7] by fitting the Marchenko-Pastur (MP) curve to the eigenvalue distribution of the signal covariance matrix and then removing noisy components. Ma and colleagues[8] use a combination of variance-stabilizing transformation (VST), low-rank matrix recovery, and exact unbiased inverse VST (EUIVST) to denoise magnitude MR signals without violating the Gaussian noise assumption of RMT. In a few studies,[9,10] complex-valued data with Gaussian noise are denoised without needing Rician correction. While effective, these techniques neglect the fact that MR data acquired with multi-channel RF

This is an open access article under the CC BY license (http://creativecommons.org/licenses/by/4.0/).

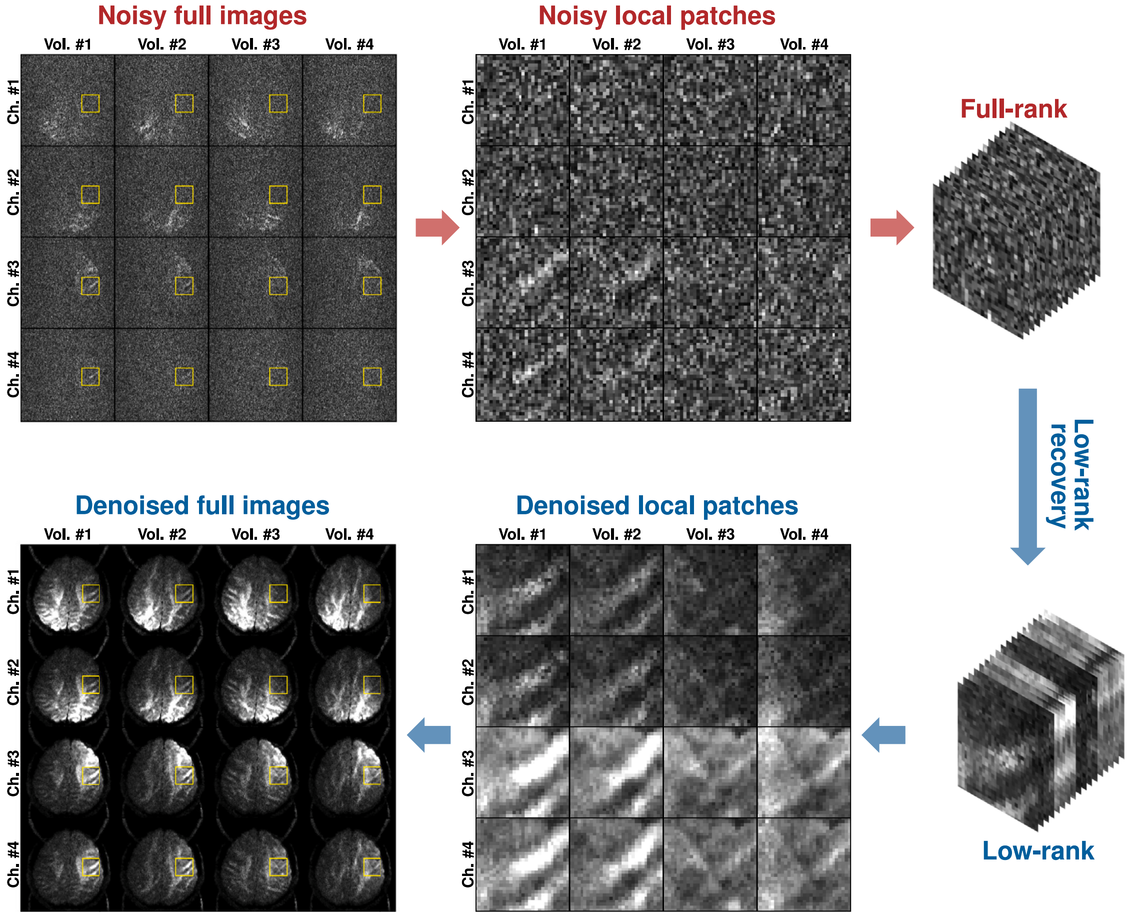

Figure 1. Redundancy and denoising
Redundancy of measurements across channels and volumes, particularly at the patch level, can be harnessed for effective denoising. Patches are extracted, stacked, and reshaped for noise removal via low-rank recovery.

receiver coils contain highly correlated information, which is useful for the separation of signal and noise.

Here, we introduce a denoising framework for effective signal recovery based on multi-channel complex (MCC) dMRI data. Inspired by RMT,[11] we exploit the often overlooked information redundancy across multiple receiver channels for effective noise removal (Figure 1). The receiver channels capture different but correlated information of an object.[12] Leveraging the Gaussian noise nature of complex data,[9] we show that a synergistic combination of channel decorrelation, background phase removal, and optimal shrinkage of singular values can significantly improve noise removal (Figure 2). Qualitative and quantitative validations using both *in silico* and *in vivo* data, covering different aspects of dMRI analysis, support the efficacy of our approach.

RESULTS

Efficacy on high-resolution *in vivo* human brain data

With a 1 mm isotropic resolution *in vivo* human brain dataset, we assessed improvements in terms of SNR, quality of diffusion-weighted images (DWIs), and estimation accuracy of tissue microstructure and axonal orientations.

DWIs

MCC denoising is remarkably effective in recovering signal contrasts buried under the noise floor (Figure 3). Magnitude denoising is ineffective in removing noise, especially at high diffusion weighting, as evidenced by the high intensity values, after denoising, in the background, where no signal is expected. In contrast, denoising with optimal shrinkage singular value decomposition (OS-SVD) using nuclear norm (*Nuc*) yields the best results with clean backgrounds. Residuals, computed as voxel-wise differences between a noisy image and its denoised counterpart, can be inspected to verify that no structural information is removed. MP-PCA using *dwidenoise* from MRtrix3,[6] called *Mag MP-PCA* from here on, removes structural details, especially in the non-DWIs. Other methods produce residual maps that show minimal to no loss of structural information. Magnitude denoising improves the SNR by, at best, 3 times, whereas MCC denoising improves the SNR by at least 5 times and, at best, 9 times. While some strategies are better than others in removing noise in the background, MCC denoising offers a significant step up from magnitude denoising in lowering the noise level, with *Nuc* performing the overall best (Figure 3, last column).

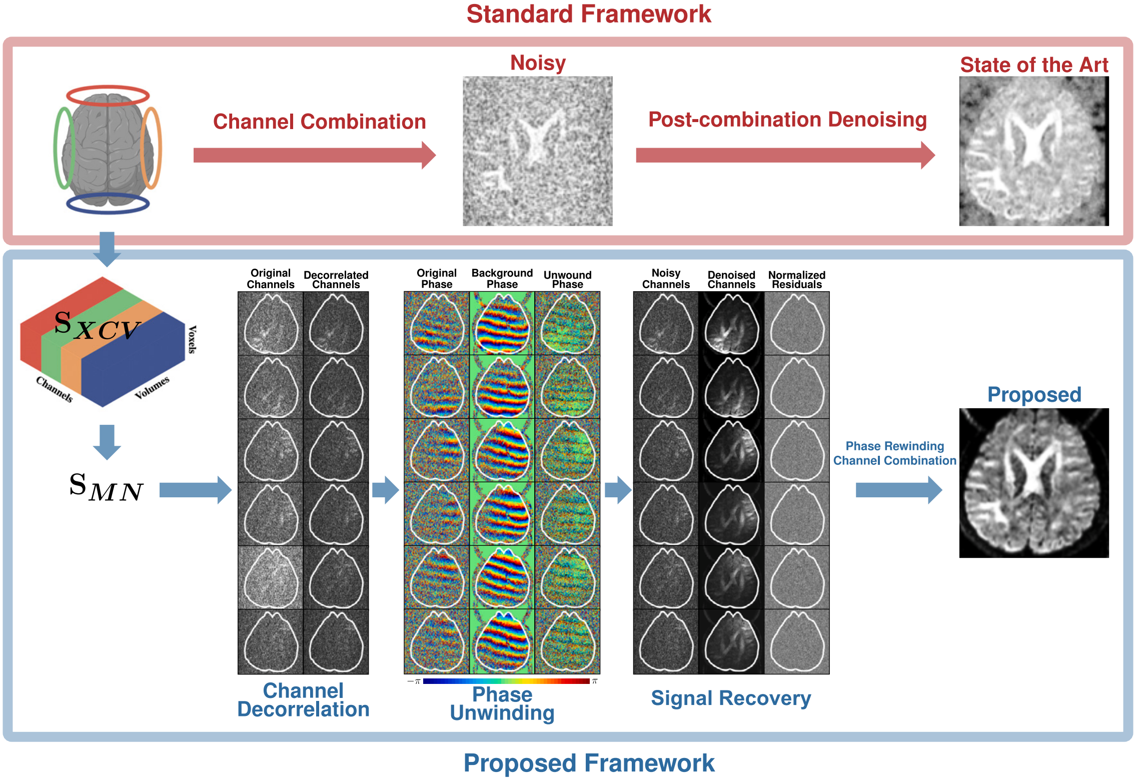

Figure 2. OS-SVD denoising framework

The proposed framework (blue) harnesses multi-channel information, removes background phase contamination, and utilizes optimal shrinkage signal recovery for significantly better restoration of image details than the standard framework (red).

Microstructure

MCC denoising outperforms magnitude denoising in improving the estimation of microstructural indices (Figure 4), including fractional anisotropy (FA), mean kurtosis (MK),[13] microscopic FA (μFA),[14] and intra-cellular volume fraction (ICVF),[15] giving more biological meaningful maps of tissue microstructure. Specifically, results given by MCC denoising exhibit good separation of white matter, gray matter, and cerebrospinal fluid (CSF) and clear typical microstructural characteristics seen in previous studies[13–16]: high FA and ICVF in white matter, where diffusion is directionally restricted by tissue microstructure, and low FA in gray matter and CSF. Magnitude denoising results in noisy FA and hyper-intense MK and almost unusable ICVF contrast, obscuring structural details at the center of the brain. The benefit of denoising is most notable from the MK and ICVF maps, as these indices are more sensitive to noise.[13,15] Improvement in μFA is less noticeable because it is estimated based on the spherical mean computed over different gradient directions[14] and is thus more robust to noise.

Axonal orientations and tractography

Compared to magnitude denoising, MCC denoising yields cleaner orientations with greater coherence and hence better delineation of fiber bundles (Figures 5 and 6). Specifically, using a ball-and-stick model,[17] MCC denoising improves the estimation of fiber orientations in, for example, a region where the corona radiata, the corpus callosum (CC), and the superior longitudinal fasciculus (SLF) interdigitate (Figure 5, white circles).[18] The orientations associated with the CC and the SLF cannot be estimated from the noisy and magnitude-denoised data due to noise (Figure 5, third and fourth rows). Using constrained spherical deconvolution,[19] OS-SVD, especially *Nuc*, produces cleaner and less random axonal orientations, especially where fibers branch (Figure 6, second row, white arrows) and reach the cortex (Figure 6, third row, white arrows). In contrast, the noisy data and other MCC denoising methods result in more random and incoherent orientations. With better axonal orientation estimates from the MCC-denoised data, tractography with the iFOD2 algorithm[20] is able to generate more biologically meaningful tractograms with less spurious segments compared to noisy and magnitude-denoised data (Figure 6, fourth and fifth rows).

Efficacy on *in silico* data

MCC versus magnitude denoising

MCC denoising outperforms magnitude denoising in recovering structural details and image contrasts with clean background even at high diffusion weighting (Figure 7). This is confirmed by the lower prediction error with respect to the ground truth (Figure 8). Among MCC denoising methods, *Nuc* yields the highest peak SNR (PSNR) and the least prediction error, with improvements particularly apparent at high diffusion weighting and at the center of the phantom with the most severe noise. In line with the literature,[21,22] while magnitude denoising can partially recover the contrast, MCC denoising is more effective by taking full advantage of the inherent but often overlooked redundant

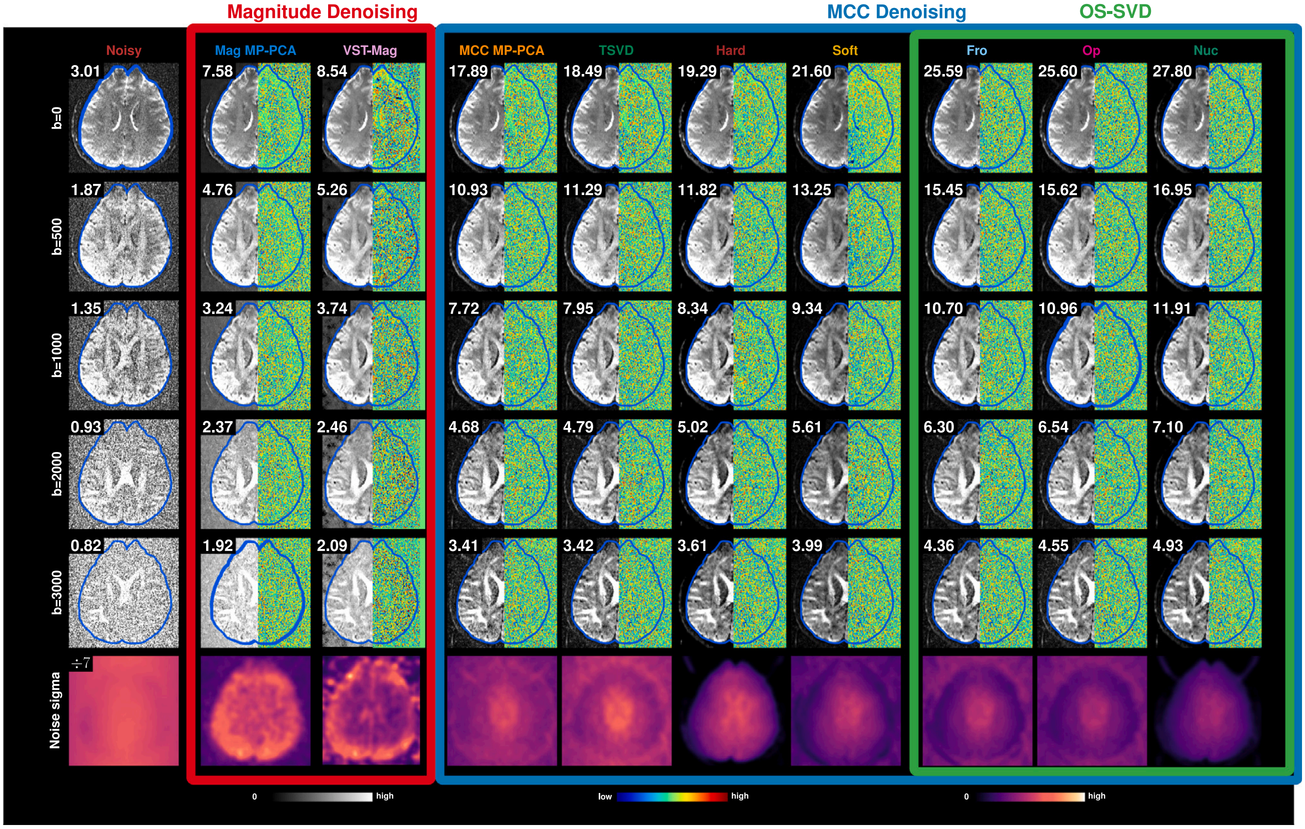

Figure 3. High-resolution *in vivo* data

Noisy and denoised diffusion-weighted images for different *b* values. The number at the top left corner of each image is the average SNR calculated across voxels within the blue contour. The right half of each image shows the normalized residuals between noisy and denoised data. The last row shows the estimated noise maps before and after denoising. All noise maps have the same scale, except for the one from noisy data, which is divided by 7 for visualization. OS-SVD increases the SNR up to 9 times without removing structural information.

information across multiple channels. This is supported by the fact that the MCC-denoised signal is more similar to the ground truth as more channels and volumes are available (Figure 8). Magnitude denoising does not benefit in the same way, despite the SNR improvement of the noisy magnitude data with the increasing number of channels.

Noise floor reduction

MCC denoising is more effective at reducing the noise floor than magnitude denoising. The effects of denoising on the noise floor can be investigated by studying the free-water signal from the CSF-like regions in the phantom (Figure S1). With high diffusivity, the signals in these regions decay rapidly with diffusion weighting and become unmeasurable under the high Rician noise floor. MCC denoising reduces the noise floor by at least 5 times compared to the noisy data and by 4 times compared to magnitude denoising, yielding a signal curve with the expected exponential decay (Figure 9). *Nuc* reduces the noise floor by at least 8-fold, yielding results that are the closest to the ground truth. MCC denoising performance is improved with more channels and volumes, further lowering the noise floor and reducing the differences between the denoised signal and the ground truth (Figures S2–S4). Magnitude denoising does not follow the same trend.

Axonal orientations and tractography

Noise causes spurious orientation estimates (Figure 10) and erroneous tractograms (Figure 11, third column). Denoising reduces false positive orientations, removes "ghost" fiber segments (Figure 11, white arrows), and yields tractograms that are closer to the ground truth (Figure 11). Magnitude denoising methods, i.e., *Mag MP-PCA* and VST with optimal shrinkage of singular values (*VST-Mag*), are not as effective in noise removal and lead to false positive orientations in regions with isotropic diffusion (Figures 10, last row, and 11, yellow arrows) and false negative orientations in regions where fibers cross (Figures 10, third column, and 11, blue arrows). MCC denoising, particularly OS-SVD approaches, yields orientations that agree substantially better with the ground truth than *Mag MP-PCA* and *VST-Mag*. With MCC denoising, tractography with the iFOD2 algorithm[20] recovers at least 21 and up to 25 fiber bundles out of a total of 27. For reference, 26 bundles are recoverable from the noise-free data, 17 from the noisy data, and 16 via *Mag MP-PCA*. MCC denoising (Table S1) results in high percentages of valid connections (VCs), low percentages of invalid connections (ICs), and low percentages of no connections (NC). *Nuc* is the overall top-performing method.

CellPress
OPEN ACCESS

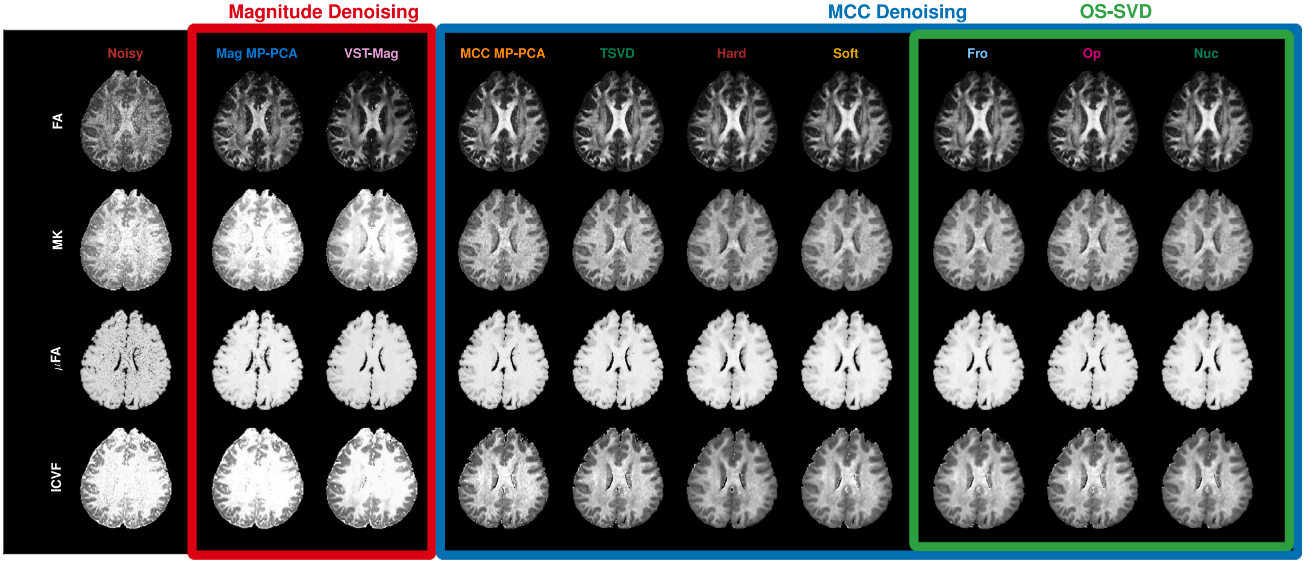

Figure 4. Microstructure

Fractional anisotropy (FA) and mean kurtosis (MK) from diffusion kurtosis imaging (DKI), microscopic FA (μFA) from spherical mean spectrum imaging (SMSI), and intra-cellular volume fraction (ICVF) from neurite orientation dispersion and density imaging (NODDI) computed for noisy and denoised data. All microstructure indices are significantly improved after denoising. In particular, improvements in MK and ICVF underscore the efficacy of MCC denoising.

DISCUSSION

In the preliminary version of this work,[23] we presented a proof of concept of using Nuc optimal shrinkage for dMRI noise reduction. Here, we presented seven denoising strategies to be used with our framework and compared them with commonly used magnitude denoising methods. With both *in silico* and *in vivo* experiments, we studied the performance of denoising strategies in terms of visual improvements, structural fidelity, noise floor reduction, and major dMRI downstream analyses including microstructure quantification, fiber orientation estimation, and tractography. Our results indicated that denoising MCC data with OS-SVD is remarkably effective at improving SNR and suppressing the noise floor in dMRI, recovering high-resolution images that would otherwise be unusable due to noise. We showed that denoising magnitude data failed to leverage information redundancy among channels, resulting in mediocre denoising outcomes. Denoising benefits a wide range of downstream analyses, allowing the quantification of tissue microstructure and the reconstruction of white matter pathways to be performed with greater accuracy. Our analyses provide insights into how the number of channels, number of volumes, background phase estimation, and noise estimation affect denoising performance. Our framework does not require any special hardware or complicated acquisition techniques but only utilizes existing MCC-valued data, which are ubiquitous in many acquisition techniques.

OS-SVD outperforms MP-PCA in MCC denoising. To study and demonstrate the advantages of OS-SVD over MP-PCA, particularly when the number of channels or volumes is limited, we generated a toy example using a noise-free matrix ($M = N =$ 20, 50, and 100) with one non-zero singular value that is associated with the signal. Introducing Gaussian noise results in small spurious eigenvalues and alters the eigenvalue corresponding to the signal. Figure 12 shows the histograms of eigenvalues before and after denoising using MP-PCA and OS-SVD *Nuc*. When the matrix size is larger, MP-PCA is effective at removing noise components with eigenvalues below a threshold. When the matrix size is small (20 × 20), MP-PCA is ineffective at separating noise from the signal component. This is due to the poor fit of the MP curve caused by the smaller number of eigenvalues. Unlike OS-SVD, denoising using MP-PCA removes noise associated with small eigenvalues but is unable to remove noise contamination in the signal, resulting in under-denoising.

Our method reshapes the signal tensor $\mathbf{S}_{XCV}$ to a 2D matrix $\mathbf{S}_{MN}$ for SVD. An alternative is to denoise the tensor directly using higher-order SVD (HO-SVD) followed by optimal shrinkage in each dimension, similar to sequentially truncated HO-SVD.[24] Briefly, with a p-th order tensor $\mathbf{S} \in \mathbb{R}^{n_1 \times n_2 \times \cdots \times n_p}$, for each $i \in \{1, \ldots, p\}$, tensor denoising applies optimal shrinkage to the i-th unfolded matrix $\mathbf{S}_{(i)} \in \mathbb{R}^{n_i \times \Pi_{k \neq i} n_k}$ of the tensor and feeds the result to the next iteration of i. Comparing tensor denoising and matrix denoising using OS-SVD with *Nuc* (Figure 13) shows no significant difference. This indicates that matrix denoising with OS-SVD (equivalent to the first iteration of tensor denoising) effectively removes noise and that subsequent iterations in tensor denoising offer little to no improvement. The tensor approach, however, might be beneficial in case of multi-TE/multi-contrast imaging.[25]

Our method can be applied to multi-band data by denoising the data after Fourier transform but before multi-band reconstruction and channel combination. The principle of utilizing the redundancy across spatial, volume, and channel dimensions remains the same.

Instead of magnitude data, denoising of complex-valued data has been shown to be more effective.[9,10,26] Our method further improves denoising by leveraging additional information from

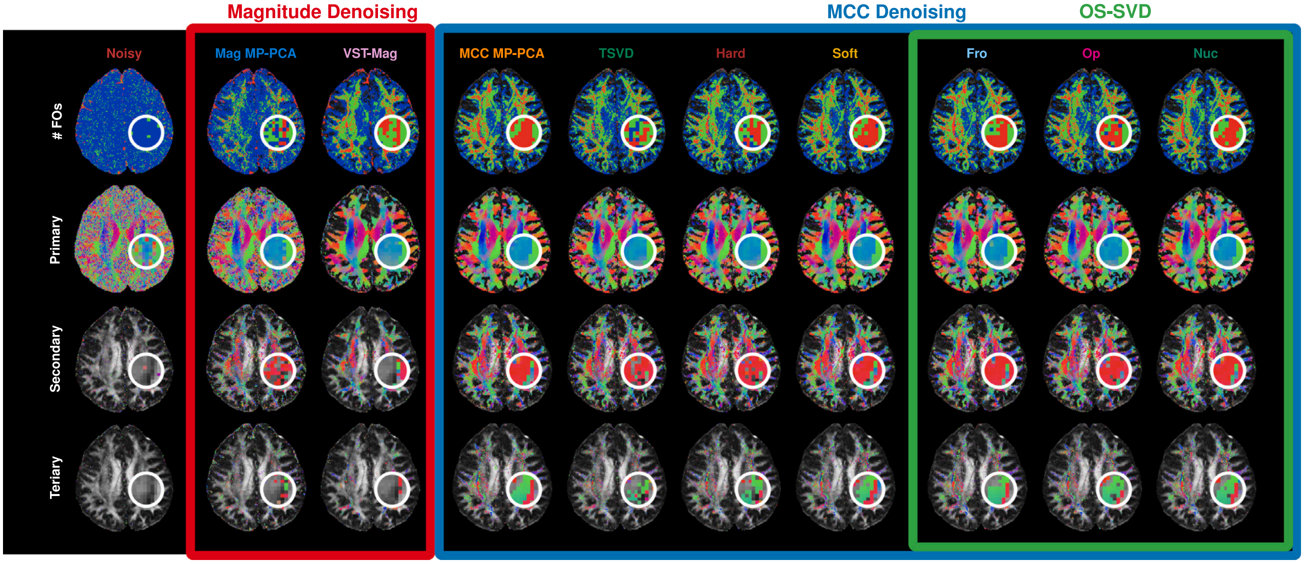

Figure 5. Detectability of fiber orientations (FOs)

The first row shows the number of detected FOs (blue: 1; green: 2; red: 3) per voxel estimated from noisy and denoised data. The next three rows show the primary, secondary, and tertiary orientations (in the order of decreasing volume fractions) shown as RGB color-coded maps (red: left-right; green: anterior-posterior; blue: inferior-superior) for orientations with volume fractions of at least 0.05. Closeups marked with white circles show where the corona radiata (primary, inferior-superior), the CC (secondary, left-right), and the SLF (tertiary, anterior-posterior) intersect. The orientations of the three bundles can be estimated correctly from the MCC-denoised data but not the noisy data and magnitude-denoised data.

multiple channels. Figure 14 further illustrates this point, showing that denoising performance improves going from magnitude data to complex data (using NORDIC[10]) and then MCC data. Our method yields 2× SNR improvement over NORDIC, giving substantially clearer FA and MK maps and better tractography, with more tracts reaching the cortex.

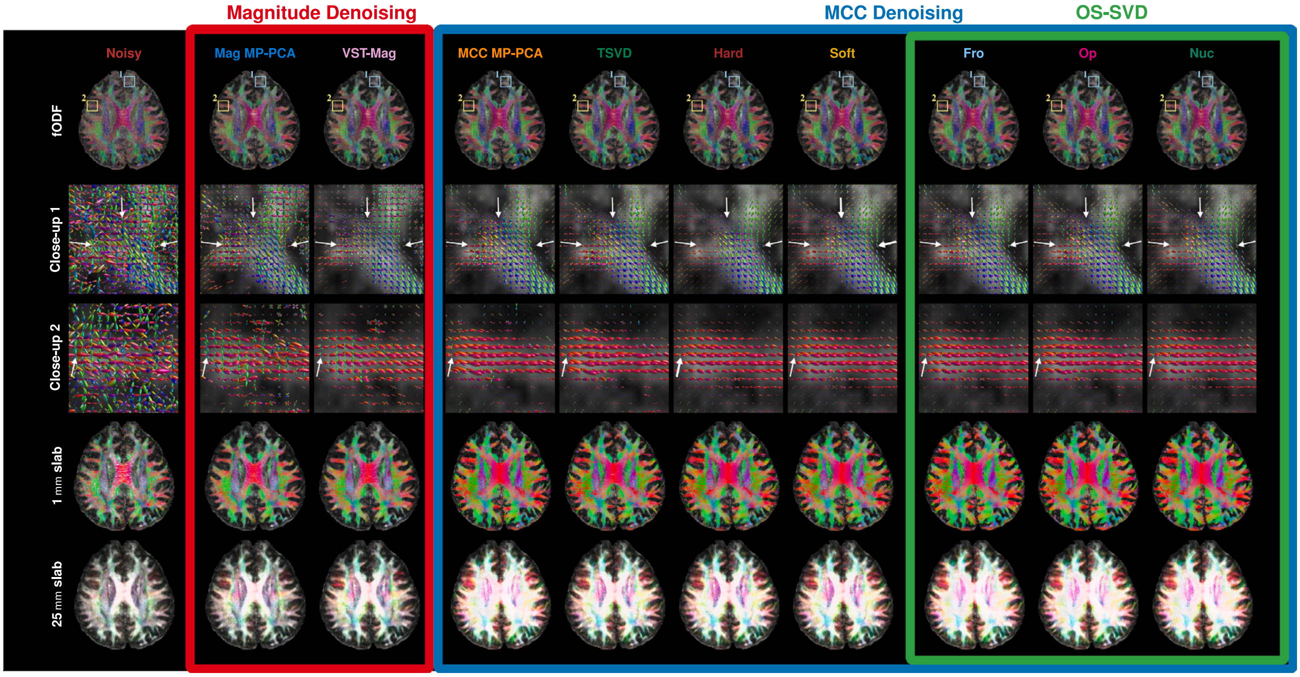

Figure 6. Axonal orientations and tractograms

Whole-brain (first row) fiber orientation distribution functions (fODFs) estimated from noisy and denoised data. Close-up views (second and third rows) show the advantages of MCC denoising over magnitude denoising, with the former giving more coherent axonal directions. The white arrows highlight the improvements given by OS-SVD over other methods. Tractograms shown for 1 (fourth row) and 25 mm (fifth row, tract opacity decreased for clarity) axial slabs confirm the advantages of MCC denoising with less spurious and more anatomically meaningful tracts not obtainable with noisy and magnitude-denoised data.

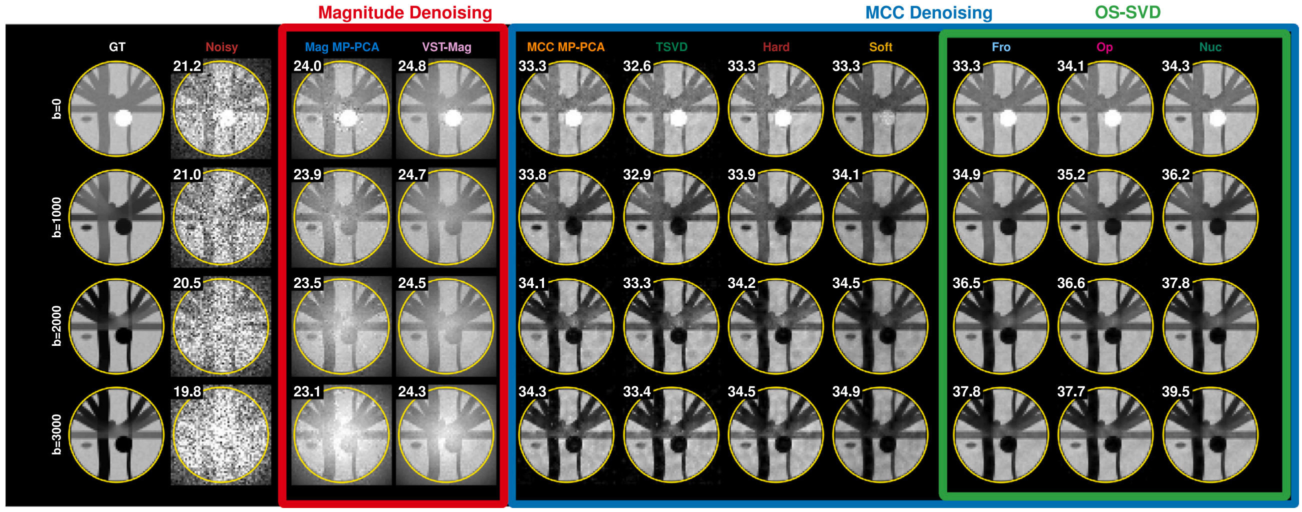

Figure 7. ***In silico* simulations**

Different denoising results from data generated via Phantomas simulations. The peak SNR (PSNR) in decibels, calculated within the yellow circle, is given at the top left.

Efforts in denoising are mainly focused on magnitude data due to its wide availability. We have demonstrated that substantial improvement in SNR can be achieved by denoising MCC-valued data with proper phase handling and optimal signal recovery, enabling high-resolution dMRI within feasible scan times. The utility of our framework is not limited to dMRI and can be applied to, for example, time series in functional MRI. We demonstrated as a proof of concept the utility of our framework in fast spiral ^{19}F lung MRI.[27,28]

Limitations of the study

Our framework has several limitations. First, MCC data needed by our framework are not always available, unlike channel-combined magnitude data. However, the non-Gaussian nature of magnitude data is dependent on how channel data are combined and hence complicates noise removal. This nature needs to be taken into account to achieve optimal denoising.[9,21] Our method allows data from multiple channels to be denoised and then combined for flexible reconstruction.

Second, MCC denoising requires greater memory and computational resources since MCC data are much larger than magnitude data. Memory requirement can be reduced by sequential block processing at the expense of speed. For example, our implementation takes 2 h to remove noise in our *in vivo* data with a 3.6 GHz Intel machine. Speedup can be achieved using a stride of $t > 1$ by skipping $(t - 1)$ voxels during block sliding. A stride of $t > 1$ in each dimension lowers the processing time by a factor of $2^{3(t-1)}$ but will potentially cause blocking artifacts. If more memory is available, blocks can be processed simultaneously to improve speed.

Third, potential misalignment of image volumes can reduce information overlap, diminishing the redundancy needed for effective denoising. To correct for misalignment, instead of using blocks from the same location, blocks from different locations in each volume can be matched and stacked for denoising, akin to block matching and 3D filtering.[29]

Lastly, the methods compared in this work rely on data redundancy. However, redundancy might be sometimes limited, resulting in data that are not necessarily low rank.[30] Data can be transformed to a high-dimensional Hilbert space for greater redundancy to improve denoising,[30] given the appropriate transformation kernel and inverse-transform parameters. Unlike Ramos-Llordén et al.,[30] our framework harnesses information from multiple channels for greater redundancy even with few gradient directions. Note that non-local block matching can be employed to increase redundancy by agglomerating similar blocks within and between volumes.

EXPERIMENTAL PROCEDURES

Resource availability

Lead contact

Code and simulated data are publicly available.[31] Further information and requests for *in vivo* data should be directed to and will be fulfilled by the lead contact, Prof. Pew-Thian Yap (ptyap@med.unc.edu).

Materials availability

The study did not generate new unique reagents.

Data and code availability

High-resolution *in vivo* data are available upon request. Code and simulated data are available at https://osf.io/f384h/.[31]

Problem formulation

Diffusion MRI measurements are acquired with multiple coil channels, imaging voxels, gradient directions, and gradient strengths. Measurement redundancy can be leveraged for effective denoising.[6] Specifically, a signal tensor $\mathbf{S}_{XCV}$, formed by voxels in a local block covering X spatial neighbors, C channels, and V volumes, can be rearranged as an $M \times N$ matrix $\mathbf{S}_{MN}$ with $M \le N$ and $M \times N = X \times C \times V$. Due to correlated measurements, the matrix has a degree of freedom that is less than XCV and is hence intrinsically low rank.[11] With random thermal noise, the matrix becomes full rank. The noise removal problem can therefore be seen as low-rank signal matrix recovery from a full-rank noisy matrix (Figure 1).

Low-rank matrix recovery

We evaluated five types of low-rank matrix recovery strategies. These methods are based on the SVD of $\mathbf{S}_{MN}$:

$$\mathbf{S}_{MN} = \sqrt{N}\mathbf{U}\Lambda\mathbf{V}^{\top}, \qquad \text{(Equation 1)}$$

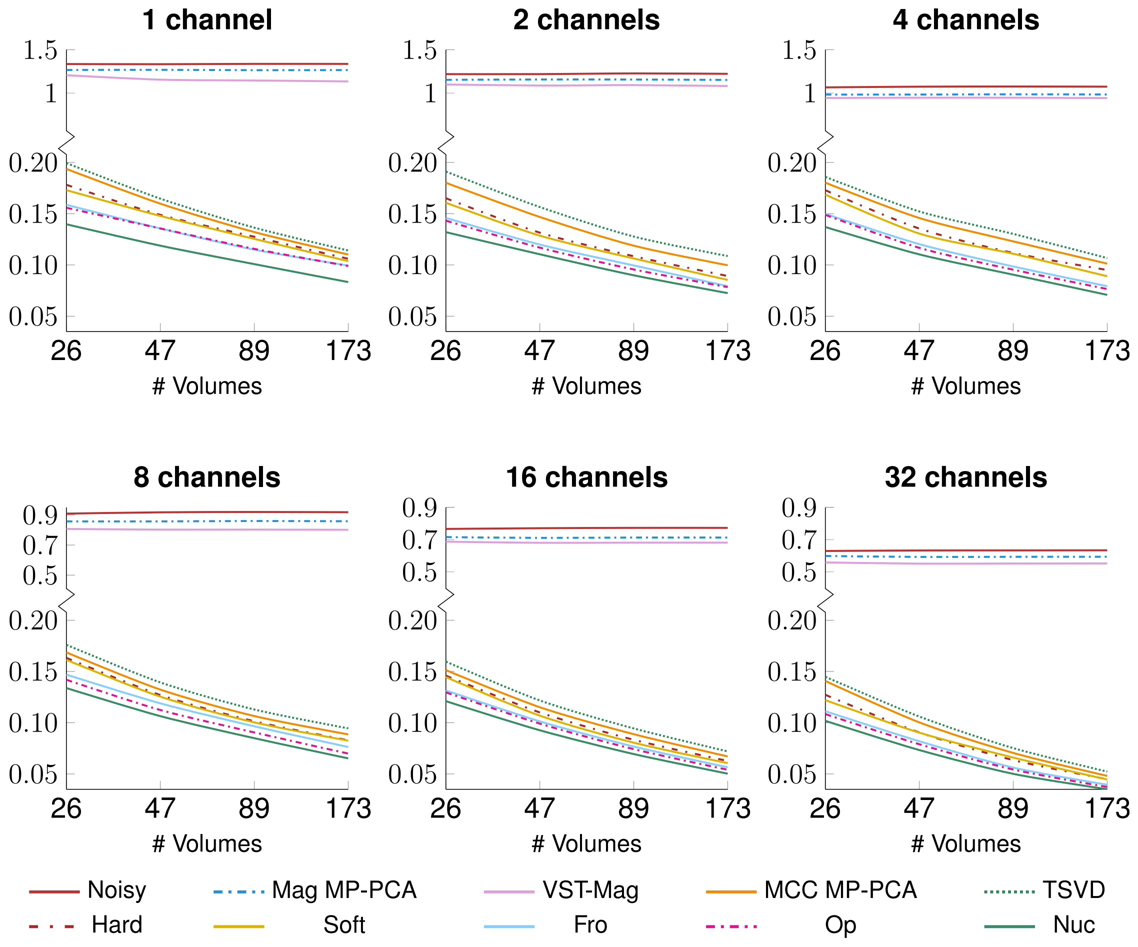

Figure 8. Prediction error
The mean normalized difference between the prediction and the ground truth (GT), computed with respect to the number of volumes and the number of channels.

or covariance matrix $\mathbf{K_S}$:

$$\mathbf{K_S} = \mathbf{S}_{MN}\mathbf{S}_{MN}^{\top} = \mathbf{U}\boldsymbol{\Sigma}\mathbf{U}^{\top}, \qquad \text{(Equation 2)}$$

where $\mathbf{U}$ and $\mathbf{V}$ are the unitary matrices containing the left and right singular vectors of $\mathbf{S}_{MN}$ and the elements of diagonal matrix $\boldsymbol{\Lambda}$ are the singular values $s_1 \geq s_2 \geq \cdots \geq s_{M-1} \geq s_M$. The elements of diagonal matrix $\boldsymbol{\Sigma}$ are the eigenvalues $\lambda_i = s_i^2, i = 1,\ldots,M$, of $\mathbf{S}_{MN}$.

MP-PCA

MP-PCA is based on the idea that for a random matrix $\mathbf{S}$ with constant noise level σ, the eigenvalues of covariance matrix $\mathbf{K_S} = \mathbf{S}\mathbf{S}^{\top}$ follow the MP distribution[11]

$$p(\lambda|\sigma,\gamma) = \begin{cases} \dfrac{\sqrt{(\lambda_+ - \lambda)(\lambda - \lambda_-)}}{2\pi\gamma\lambda\sigma^2} & \text{if } \lambda_- \leq \lambda \leq \lambda_+, \\ 0 & \text{otherwise,} \end{cases} \qquad \text{(Equation 3)}$$

where $\lambda_{\pm} = \sigma^2(1 \pm \sqrt{\gamma})^2$ with $\gamma = \frac{M-P}{N}$, and $P < M$ is the number of signal components. The threshold P can be estimated simultaneously with σ based on the procedure described by Veraart et al.[11] Only components with eigenvalues larger than a threshold are retained:

$$\hat{\lambda}_i = \begin{cases} \lambda_i & \lambda_i \geq (M-P)\hat{\sigma}^2(P), \\ 0 & \text{otherwise,} \end{cases} \qquad \text{(Equation 4)}$$

where

$$\hat{\sigma}^2(P) = \frac{\lambda_{P+1} - \lambda_M}{4\sqrt{\gamma}}. \qquad \text{(Equation 5)}$$

The noise-free signal matrix $\hat{\mathbf{S}}$ is recovered as

$$\hat{\mathbf{S}} = \sqrt{N}\mathbf{U}\hat{\boldsymbol{\Lambda}}\mathbf{V}^{\top}, \qquad \text{(Equation 6)}$$

where $\hat{\boldsymbol{\Lambda}}$ is a diagonal matrix with elements $\hat{s}_i = \sqrt{\hat{\lambda}_i}, i = 1,\ldots,M$.

OS-SVD

OS-SVD optimally shrinks $s_1 \geq s_2 \geq \cdots \geq s_{M-1} \geq s_M$ according to a cost function, giving the following advantages over MP-PCA:

- OS-SVD does not just zero out $M - P$ singular values like MP-PCA but instead manipulates all singular values s_i to mitigate noise contamination. This is especially important when M is small (e.g., due to limited channels, volumes, or block size) because limited singular values are available for accurate MP-PCA.
- OS-SVD is proven to be optimal with respect to a cost function.[32]

Letting $z = z(y) = \frac{1}{\sqrt{2}}\sqrt{y^2 - \delta - 1 + \sqrt{(y^2 - \delta - 1)^2 - 4\delta}}$ when $y \geq 1 + \sqrt{\delta}$ and 0 otherwise, the shrinkage functions $\eta^{*}(s)$ for minimizing the Frobenius norm (*Fro*) $\| \mathbf{S} - \hat{\mathbf{S}} \|_F$, the nuclear norm (*Nuc*) $\| \mathbf{S} - \hat{\mathbf{S}} \|_*$, and the operator norm (*Op*) $\| \mathbf{S} - \hat{\mathbf{S}} \|_{\text{op}}$ are, respectively,

CellPress
OPEN ACCESS

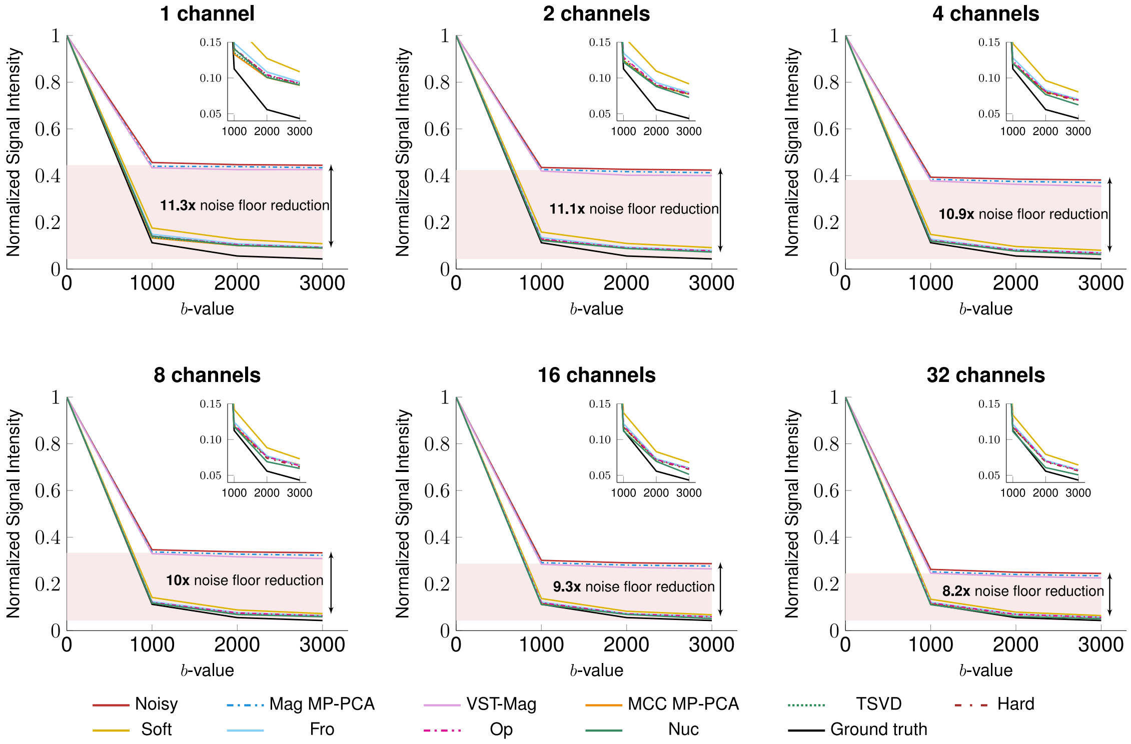

Figure 9. Free-water diffusion

Normalized free-water diffusion signal from GT, noisy, and denoised data with 173 volumes. Shaded regions indicate the original noise floor. The zoomed-in plots highlight the differences between MCC methods.

$$\eta^{\mathrm{Fro}}(s) = \begin{cases} \frac{1}{y}\sqrt{\left(y^2 - \delta - 1\right)^2 - 4\delta} & y \geq 1+\sqrt{\delta}, \\ 0 & \text{otherwise}, \end{cases} \quad \text{(Equation 7)}$$

$$\eta^{\mathrm{Nuc}}(s) = \begin{cases} \frac{1}{z^2 y}\left(z^4 - \delta - \sqrt{\delta}yz\right) & z^4 \geq \delta+\sqrt{\delta}yz, \\ 0 & \text{otherwise}, \end{cases} \quad \text{(Equation 8)}$$

and

$$\eta^{\mathrm{Op}}(s) = z, \quad \text{(Equation 9)}$$

where $y = s/\sigma$ with noise level σ and $\delta = M/N$. The noise-free matrix is estimated as

$$\widehat{\mathbf{S}} = \sqrt{N}\sigma \mathbf{U}\widehat{\mathbf{\Lambda}}\mathbf{V}^T, \quad \text{(Equation 10)}$$

with diagonal matrix $\widehat{\mathbf{\Lambda}}$ containing elements $\eta^{\cdot}(s_1), \eta^{\cdot}(s_2), \ldots, \eta^{\cdot}(s_M)$.

Truncated SVD (TSVD)

TSVD estimates a low-rank matrix by keeping only singular values larger than a threshold and recovers the signal with Equation 10. A possible choice for the threshold is $\sigma(1+\sqrt{\delta})$,[33] which results in shrinkage

$$\eta^{\mathrm{TSVD}}(s) = \begin{cases} y & y \geq 1+\sqrt{\delta}, \\ 0 & \text{otherwise}. \end{cases} \quad \text{(Equation 11)}$$

Hard thresholding (Hard)

Hard-thresholding shrinkage[34] is realized using

$$\eta^{\mathrm{Hard}}(s) = \begin{cases} y & y \geq y^{\dagger}, \\ 0 & \text{otherwise}, \end{cases} \quad \text{(Equation 12)}$$

where the threshold $y^{\dagger}$ is calculated as

$$y^{\dagger} = \sqrt{2(\delta+1)+\frac{8\delta}{\delta+1+\sqrt{\delta^2+14\delta+1}}}. \quad \text{(Equation 13)}$$

Note that hard thresholding is a form of TSVD but with a provably optimal threshold. Signal is recovered with Equation 10.

Soft thresholding (Soft)

Soft-thresholding shrinkage not only discards small singular values but also alters the retained singular values.[35–37] It is realized with shrinkage

$$\eta^{\mathrm{Soft}}(s) = \begin{cases} y - \left(1+\sqrt{\delta}\right) & y \geq 1+\sqrt{\delta}, \\ 0 & \text{otherwise}. \end{cases} \quad \text{(Equation 14)}$$

Similar to *Hard*, the noise-free signal matrix can be recovered with Equation 10. These shrinkage strategies can be categorized as

(1) Removing singular values below a threshold and retaining the rest, with the threshold depending only on the matrix size and noise level (*TSVD* and *Hard*),
(2) Removing singular values below a threshold and retaining the rest, with the threshold depending on the matrix size and the singular values (MP-PCA), or
(3) Altering all singular values (*Soft*, *Fro*, *Op*, and *Nuc*).

Noise estimation

Accurate estimation of the noise level σ is key to effective shrinkage. In MP-PCA, σ is estimated simultaneously with P[11]:

$$\widehat{\sigma} = \sqrt{\frac{\sum_{i=P+1}^{M}\lambda_i}{M-P}}. \quad \text{(Equation 15)}$$

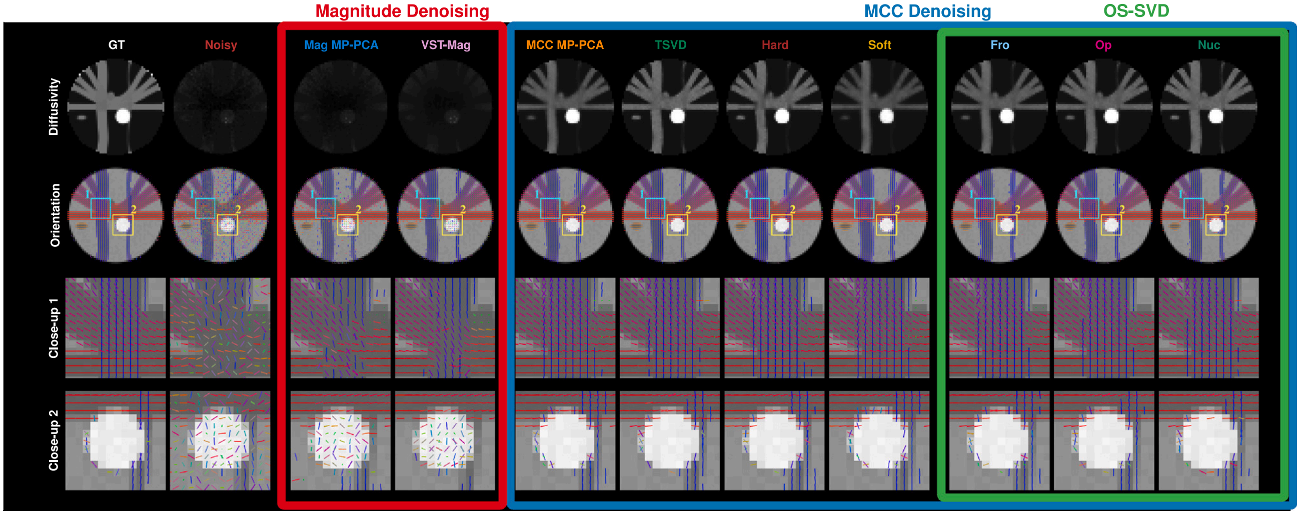

Figure 10. Detection of FOs

FO estimation using *bedpostX* from GT, noisy, and denoised data. Top row: longitudinal diffusivity estimated from the ball-and-stick model, shown in grayscale ranging from 0 (black) to 3.0×10^{-3} mm^2 s^{-1} (white). Second row: RGB color-coded FO maps (red: left-right; green: anterior-posterior; blue: inferior-superior), shown only for orientations with volume fractions of at least 0.05. The last two rows show close-up views of the second row. Magnitude denoising causes incorrect diffusivity (first row), missing and incorrect orientations in a fiber-crossing region (third row), and false positive orientations in an isotropic region (last row). In contrast, MCC denoising results are strikingly similar to the GT.

This approach is later adopted in a variance-stabilization framework[8] to Gaussianize Rician noise prior to denoising. Alternatively, we can estimate σ with[32]

$$\hat{\sigma} = \frac{s_{\frac{1}{2}}}{\sqrt{N \cdot \delta_{\frac{1}{2}}}}, \qquad \text{(Equation 16)}$$

where $s_{\frac{1}{2}}$ is the median empirical singular value of $\mathbf{S}$ and $\delta_{\frac{1}{2}}$ is the median of the MP distribution determined by solving for μ in

$$\int_{\delta_-}^{\mu} \frac{\sqrt{(\delta_+ - \nu)(\nu - \delta_-)}}{2\pi\nu} d\nu = 0.5, \delta_- \le \mu \le \delta_+, \qquad \text{(Equation 17)}$$

where $\delta_{\pm} = (1 \pm \sqrt{\delta})^2$. Empirical results (Figure S5) indicate that estimator (Equation 16) yields greater accuracy. We therefore use this estimator for all MCC denoising methods, except for *MCC MP-PCA*, which concurrently estimates the noise level and removes noise based on MP-PCA with MCC data.

Determining the matrix size

The size of the signal matrix, $M \times N$, affects shrinkage. Generally, we want M to be as large as possible for a better estimate of the distribution of the singular values. This can be achieved via a larger block size, more channels, and more image volumes. When acquiring sufficient volumes with sufficient channels is not possible, one can resort to using a larger block size to increase M, which, however, comes at the cost of redundancy. Larger blocks are less similar than smaller blocks. In light of this, we suggest using an isotropic 3D block with length set to the smallest odd integer $k \ge \sqrt[3]{CV}$, resulting in $M = CV \le N = k^3$. Alternatively, in case of low spatial resolution, one can set k to be as small as 3 (smallest block size to leverage spatial redundancy) so that $M = \min(V, 27C)$ and $N = \max(V, 27C)$. This choice, however, requires V to be sufficiently large for effective denoising.

Automated pipeline for effective noise removal

Inspired by the work of Lemberskiy et al.,[21,22] after Fourier transform, our denoising pipeline involves (1) channel decorrelation, (2) phase unwinding, (3) noise mapping and removal, and (4) image reconstruction (Figure 2).

Channel decorrelation

RMT assumes independent and identically distributed (iid) Gaussian noise, while in fact, noise across channels is highly correlated.[38] We use Mahalanobis whitening transformation for cross-channel decorrelation:

$$\mathbf{S}_{XCV} = \sum_{c'=1}^{C} \left(\Phi^{-1/2}\right)_{cc'} \mathbf{S}_{Xc'V}, \Phi^{-1/2} = \mathbf{U}\varphi^{-1/2}\mathbf{U}', \qquad \text{(Equation 18)}$$

where Φ is the noise covariance matrix computed from channel-specific noise acquired without excitation, $\varphi = \mathbf{U}'\Phi\mathbf{U}$ is the diagonal matrix of eigenvalues of Φ, and $\mathbf{U}$ is a unitary rotation matrix. Φ can be calculated based on 1 k-space line sample with no RF excitation or channel signals of non-brain voxels.

Phase unwinding

The complex MRI signal $\mathbf{S}$ with modulus r and phase φ can be written as

$$\mathbf{S} = re^{i\varphi}. \qquad \text{(Equation 19)}$$

The phase $\varphi = \varphi_{BG} + \varphi_{noise}$ consists of random noise phase φ_{noise} and background phase φ_{BG} introduced by coil variations and physiological effects (head movements, respiratory activities, etc.).[39,40] Inspired by the work of Sprenger et al.,[41] we first denoise the real and imaginary parts of the signal using our method described in noise mapping and removal. From the denoised data, we calculate the background phase φ_{BG} to obtain the phase-unwound signal S_{corr} as

$$\mathbf{S}_{corr} \approx re^{i(\varphi - \varphi_{BG})}. \qquad \text{(Equation 20)}$$

The imaginary part of S_{corr} is now pure noise and can be discarded, leaving only the real part for subsequent steps.

Noise mapping and removal

For each voxel x, a local block is chosen from the real part of $\mathbf{S}_{corr}$ to form $\mathbf{S}_{MN}$ for denoising. The noise standard deviation σ_x is estimated as described in noise estimation. Considering that x can be associated with multiple blocks, we determine the noise-free signal $\hat{\mathbf{S}}_x$ as

$$\hat{\mathbf{S}}_x = \frac{\sum_{j=1}^{J} w_j \hat{\mathbf{S}}_j(x)}{\sum_{j=1}^{J} w_j}, \qquad \text{(Equation 21)}$$

CellPress
OPEN ACCESS

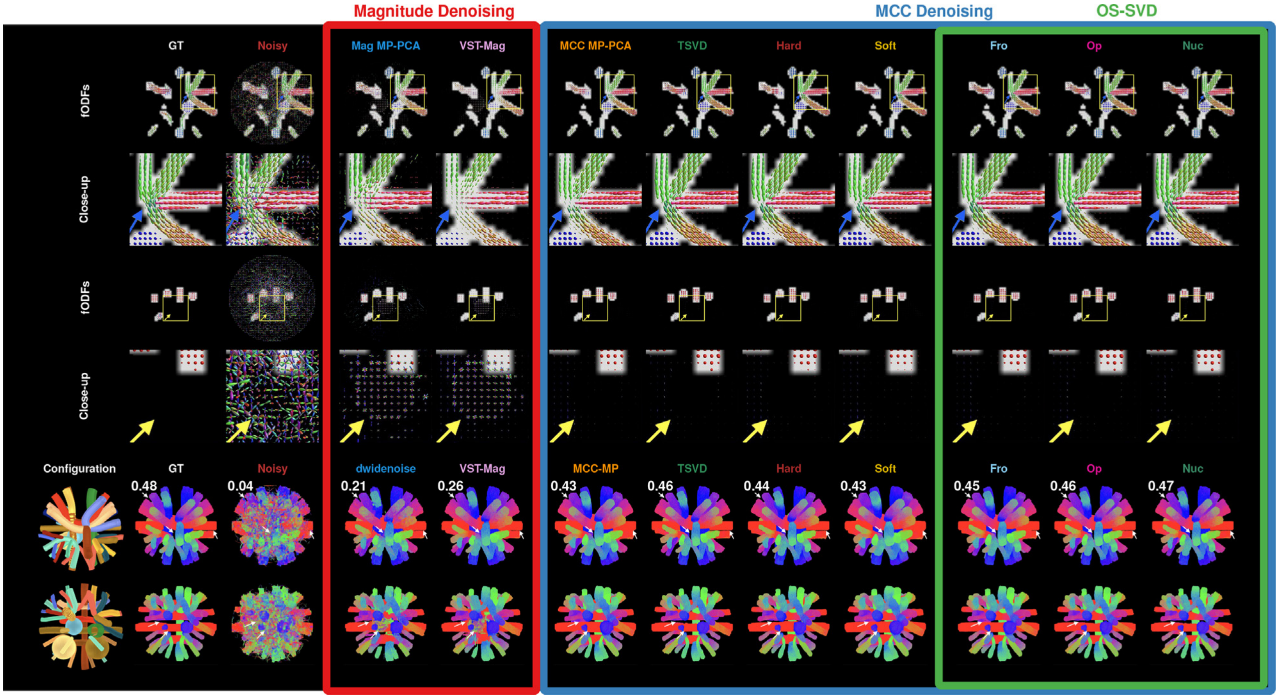

Figure 11. fODFs and tractograms

fODFs (top 4 rows) and tractograms (last 2 rows) from GT, noisy, and denoised data. Low SNR causes false negatives (missing fODF glyphs, blue arrows) and false positives (spurious glyphs, yellow arrows), particularly visible for *Noisy*, *Mag MP-PCA*, and *VST-Mag*. The tractography score of each tractogram, which ranges from 0 (low) to 1 (high), is stated at the top left corner. White arrows mark notable locations of ghost fibers: fibers that are not in the configuration but might appear in tractograms due to noise. Overall, using MCC data yields cleaner fODFs and tractograms. OS-SVD *Nuc* produces visually the best result closest to the GT with no ghost fibers.

where $\widehat{\mathbf{S}}_j(x)$ is the denoised signal of x in block j, J is the total number of blocks containing x, and w_j is the weighting factor. To reduce Gibbs ringing artifacts, a block with a lower rank is assigned a greater weight[8]:

$$w_j = \frac{1}{1+R_j}, \qquad \text{(Equation 22)}$$

where R_j is the rank of $\widehat{\mathbf{S}}_j$.

Phase rewinding (optional)

In the situation where the phase is needed for subsequent processing,[42] the phase can be rewound.

Image reconstruction

The denoised data from multiple channels can be used for parallel imaging reconstruction, multi-band imaging reconstruction, or channel combination via sum of squares or adaptively with channel sensitivity maps.[38,42–44] We used SENSE[38] for channel combination.

Evaluation approaches

We evaluated the following low-rank signal recovery strategies based on MCC data.

- MP-PCA[6] based on MCC data (*MCC MP-PCA*) inspired by Lemberskiy et al.,[21]
- *TSVD*,[33]
- *Hard*,[34]
- *Soft*,[36,37] and
- OS-SVD,[32] which optimally shrinks singular values according to
  - *Fro*,
  - *Op*, or
  - *Nuc*.

We further included for comparison two widely used approaches based on magnitude channel-combined data.

- *Mag MP-PCA*[6] and
- *VST-Mag*.[8]

These approaches are summarized in Figure 15 and Table S2, showing their differences in terms of singular value modification, noise estimation, and input type. The core signal recovery algorithms were adapted from respective studies. *VST-Mag* was based on the code provided by the authors[8] with an author-suggested 5×5×5 overlapped sliding block. As mentioned in determining the matrix size, *Mag MP-PCA* and our framework should be applied with the smallest isotropic block size $k \geq \sqrt[3]{CV}$, resulting in $M = CV \leq N = k^3$. However, for fair comparison, we fix the kernel to 5 × 5 × 5, similar to *VST-Mag*. Channel combination was performed using SENSE,[38] resulting in channel-combined magnitude data with Rician noise, satisfying the assumption of *VST-Mag*. For *Mag MP-PCA*, MP curve-fitting parameters were estimated and corrected for Rician bias as in the original paper.[11] We compared the MRtrix and Dipy[45] implementations of MP-PCA and did not find significant differences (Figure S7).

In vivo data processing and evaluation

We compared denoising efficiency using 1 mm isotropic resolution dMRI data of a consented healthy adult, collected with a 3T Siemens MAGNETOM Prisma scanner, 32-channel head coil, single-band acquisition, 4,600 ms TR, 99 ms TE, 78° flip angle, $\frac{5}{8}$ partial Fourier, 4 diffusion weightings of 500, 1,000, 2,000, and 3,000 s mm^{-2} with 5, 10, 20, and 30 non-collinear gradient directions, respectively, and a non-DWI ($C = 32$, $V = 66$). Channel combination was done using SENSE.[38]

Structure preservation and SNR

As different methods involve different steps in preprocessing the data before denoising (VST, EUIVST, channel decorrelation, and phase unwinding), for

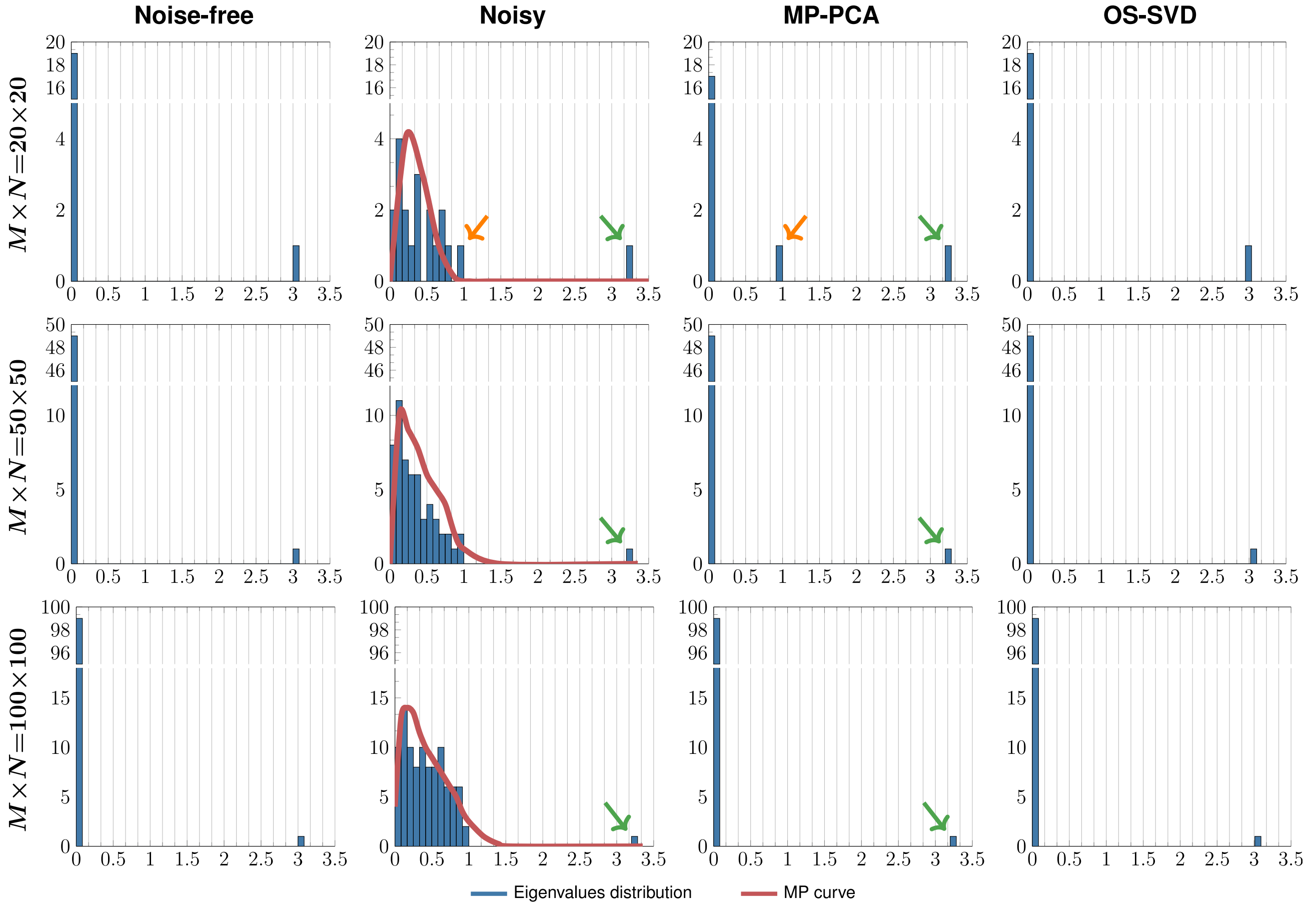

Figure 12. Effects of matrix size on denoising performance

Histograms of eigenvalues from noise-free matrices (first column), noisy matrices (second column), and denoised matrices given by MP-PCA (third column) and OS-SVD (last column). Noise not only introduces spurious eigenvalues but also alters the signal eigenvalue (green arrows). Denoising based on MP-PCA (red) simply retains all eigenvalues above the threshold and does not attempt to recover the actual eigenvalue of the signal component. When the matrix size is small (first column), the lack of eigenvalues results in an inaccurate MP curve that does not cleanly separate noise from signal, resulting in incorrect retaining of a noise component (orange arrows) after denoising. In contrast, OS-SVD removes all noise components and recovers the signal component regardless of the matrix size.

fair comparison, we evaluated the ability of each method in preserving structural information by calculating the normalized difference between the input and output of the denoising step: the noisy and denoised data for *Mag MP-PCA*, the signal after VST and before EUIVST for *VST-Mag*, and the signal before and after low-rank matrix recovery for other methods. Methods that preserve brain structures produce a Gaussian-distributed residual map with no structural information. We used the method described by Veraart et al.[11] to estimate the voxel-wise noise sigma for the noisy data and different denoising results for SNR evaluation. Using the same method[11] for SNR computation ensures a fair comparison of the different denoising strategies.

Microstructure model fitting, axonal orientation estimation, and tractography

The purpose of denoising is to produce high-quality images that can be used in subsequent analyses. We fitted microstructure models to the noisy and denoised data. Three common models were used, including diffusion kurtosis imaging (DKI),[13] spherical mean spectrum imaging (SMSI),[14,16] and neurite orientation dispersion and density imaging (NODDI).[15] Model parameters were chosen as described in the original papers. We used *bedpostX*[17] for axonal orientation quantification, multi-shell multi-tissue constrained spherical deconvolution (MSMT-CSD)[19,46] for fiber orientation distribution functions (fODF) estimation, and iFOD2[20] for tractography. The number of seeds was fixed. All data were corrected for motion and distortion[47] before the aforementioned analyses.

In silico data simulation and evaluation

We simulated noise-free diffusion MRI data using a digital phantom.[48] The phantom consisted of 27 size-varying fibers with straight, bending, fanning, kissing, and crossing configurations and 3 isotropic diffusion regions. Fibers had parallel diffusivity 1.7×10^{-3} mm^2 s^{-1} and perpendicular diffusivity 0.4×10^{-3} mm^2 s^{-1}, whereas isotropic diffusion regions had diffusivity 3.0×10^{-3} mm^2 s^{-1}, mimicking typical values in the human brain. There were 24, 48, and 96 DWIs for 4 diffusion weightings of 1,000, 2,000, and 3,000 s mm^2, respectively, and 5 non-DWIs, giving a total of 173 volumes (1 mm isotropic resolution). To quantify how the number of volumes affects denoising, we used one-eighth, one-fourth, and half of the DWIs in the 173 volume dataset to create datasets with 26, 47, and 89 volumes. To study the effects of the number of channels on denoising, we varied $C \in \{1, 2, 4, 8, 16, 32\}$. For each dataset, the multi-channel data were created by adding iid. Gaussian noise to the real and imaginary parts with the noisy signal $\tilde{S}_c(x,v)$ of voxel x, channel c, and volume v is given as

$$\tilde{\mathbf{S}}_c(x,v) = R_c(x)\mathbf{S}_c(x,v)e^{i\varphi_{\mathrm{BG}}(x,v)} + \epsilon_c^{(\mathrm{r})}(x,v) + i\epsilon_c^{(\mathrm{i})}(x,v), \quad \text{(Equation 23)}$$

where $\mathbf{S}_c(x,v)$ is the noise-free signal, $R_c(x)$ is the channel sensitivity map, and $\epsilon_c^{(\mathrm{r})}(x,v)$ and $\epsilon_c^{(\mathrm{i})}(x,v) \in \mathcal{N}(0,\sigma^2)$ are complex noise added to channel c. The background phase $\varphi_{\mathrm{BG}}(x,v)$ was simulated using a bidimensional sinusoid

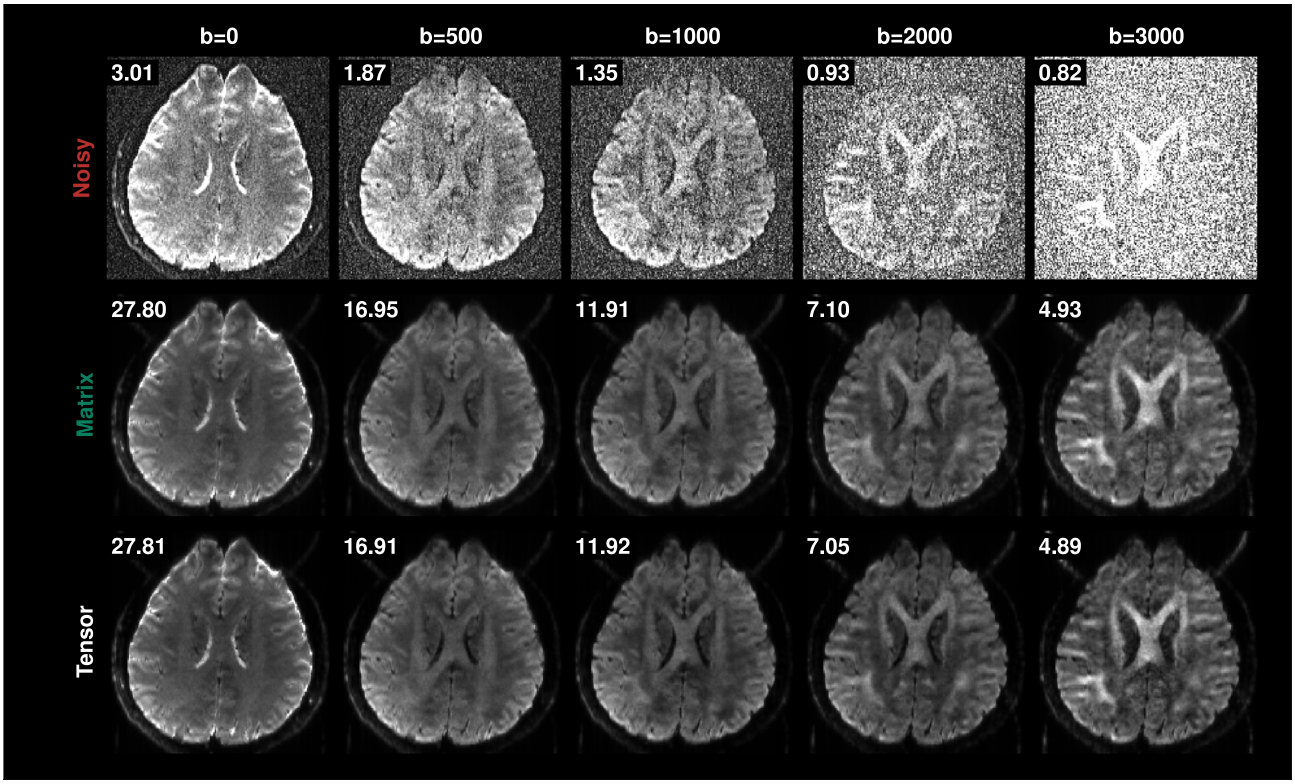

Figure 13. Tensor denoising
Noisy and denoised diffusion-weighted images given by matrix and tensor denoising for different b values. The SNR is shown at the top left corner of each image.

along the x direction and y direction, with random shift along the z direction mimicking the smooth intra-slice and abrupt interslice transitions.[40] A spatially varying noise map $\sigma(x)$ with noise levels higher at the center and lower at the periphery was employed, resulting in SNRs of 2–15. The phantom configuration, background phase, noise map, and channel sensitivity maps are shown in Figure S1.

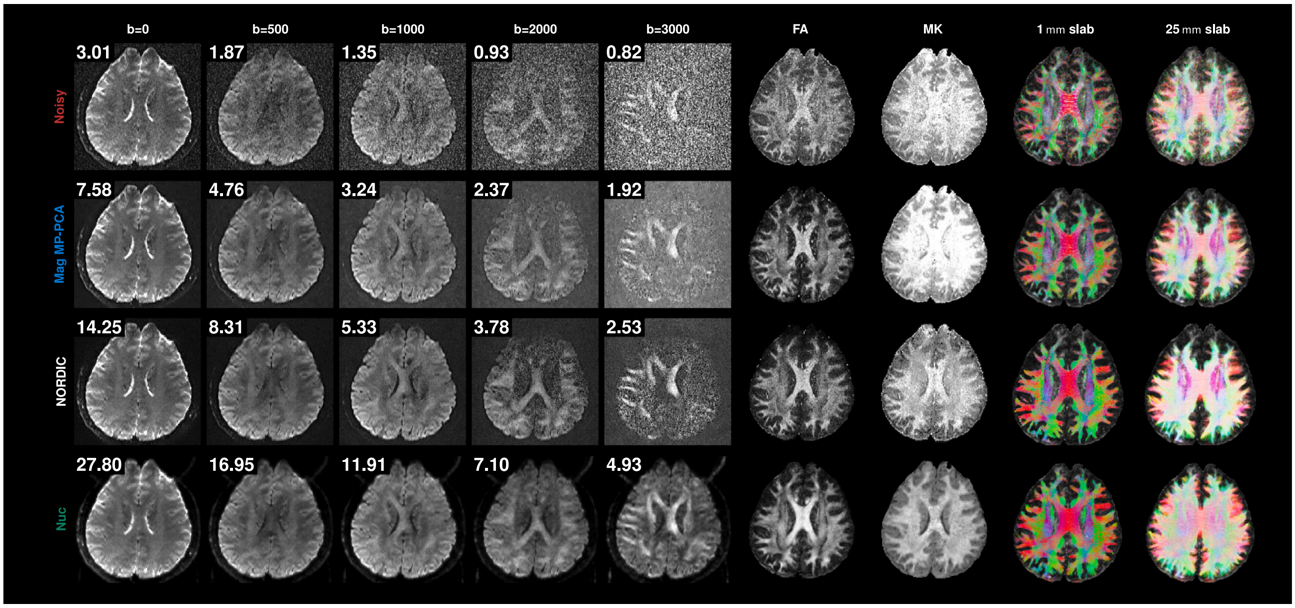

Figure 14. Magnitude, complex, and MCC denoising
DWIs, FA, MK, and tractograms from noisy data and from denoising results given by *Mag MP-PCA*, NORDIC, and *MCC-Nuc*. The number at the top left corner of each image is the average SNR calculated across brain voxels. Images in each column have the same scale.

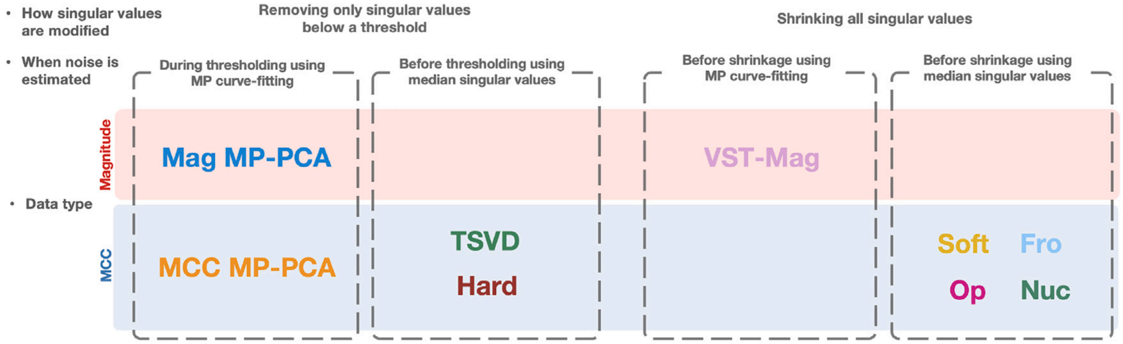

Figure 15. Summary of denoising strategies

Denoising strategies differ in terms of how the singular values are modified (thresholding versus shrinkage), when the noise level is estimated, and the type of input (channel-combined magnitude or MCC data).

Noise mapping, background phase estimation, noise floor reduction, and denoising accuracy

Noise maps for *Mag MP-PCA* and *MCC MP-PCA* were obtained using Equation 15 as described by Veraart et al.[11] *VST-Mag* estimates the Rician noise sigma for VST[49] and then the Gaussian noise sigma (after VST) for denoising.[11] For fair comparison with other methods assuming Gaussian noise, we only show the Gaussian noise map estimated by *VST-Mag*. Noise maps for *TSVD*, *Hard*, *Soft*, and OS-SVD were obtained using Equation 16 as suggested by Gavish and Donoho.[32] The outcome of Equation 16 is closest to the ground truth (Figure S5). The estimated background phases from different MCC strategies are similar to each other and the ground truth, capturing the smooth and spatially varying nature of the background phase (Figure S6). We assessed the effectiveness in noise floor reduction by comparing, before and after denoising, the signal corresponding to the highest b value in regions containing CSF. As water molecules diffuse unhindered in these regions, the signal decays exponentially with b value and is more susceptible to the noise floor. To quantify denoising accuracy, we calculated the mean-normalized difference between the denoised data and the noise-free ground truth across voxels and volumes:

$$\text{Error} = \frac{1}{XV}\sum_{x,v}^{X,V}\frac{|\widehat{\mathbf{S}}(x,v) - \mathbf{S}(x,v)|}{\mathbf{S}(x,v)}, \qquad \text{(Equation 24)}$$

where $\widehat{\mathbf{S}}(x,v)$ and $\mathbf{S}(x,v)$ are, respectively, the denoised and ground-truth images at voxel x and volume v. We also computed the PSNR[8]:

$$\text{PSNR} = 10\log_{10}\frac{1}{\text{MSE}}, \qquad \text{(Equation 25)}$$

where MSE is the mean squared error between the denoised and noise-free images. PSNR was calculated for each b value separately.

Fiber orientations, fODF estimation, and tractography

We used *bedpostX*[17] to estimate fiber orientations, MSMT-CSD[19,46] to estimate fODFs, and iFOD2[50] to generate tractograms. Tractometer[51] was used to calculate the fractions of VCs, ICs, and NCs. A perfect tractogram will result in VCs of 1 and ICs and NCs of 0. We define a tractography score that takes into account VCs, ICs, and NCs[52]:

$$\text{Score} = 1 - \frac{\sqrt{(\text{VC} - 1)^2 + \text{IC}^2 + \text{NC}^2}}{\sqrt{2}}, \qquad \text{(Equation 26)}$$

with 0 corresponding to fully ICs or NCs and 1 corresponding to fully VCs.

SUPPLEMENTAL INFORMATION

Supplemental information can be found online at https://doi.org/10.1016/j.patter.2024.100954.

ACKNOWLEDGMENTS

This work was supported in part by the United States National Institutes of Health (NIH) through grants MH125479 and EB008374. Figure 2 was created in part with BioRender.com.

AUTHOR CONTRIBUTIONS

Conceptualization, K.H. and P.-T.Y.; data acquisition, W.-T.C.; data analysis, K.H., W.-T.C., and Y.W.; manuscript writing and preparation, K.H. and P.-T.Y.; supervision, P.-T.Y. All authors reviewed the results and approved the final version of the manuscript.

DECLARATION OF INTERESTS

The authors declare no competing interests.

Received: September 12, 2023
Revised: February 20, 2024
Accepted: February 21, 2024
Published: March 14, 2024

REFERENCES

1. Gudbjartsson, H., and Patz, S. (1995). The Rician distribution of noisy MRI data. Magn. Reson. Med. *34*, 910–914.
2. Budinger, T.F., Bird, M.D., Frydman, L., Long, J.R., Mareci, T.H., Rooney, W.D., Rosen, B., Schenck, J.F., Schepkin, V.D., Sherry, A.D., et al. (2016). Toward 20 T magnetic resonance for human brain studies: opportunities for discovery and neuroscience rationale. Magnetic Resonance Materials in Physics, Biology and Medicine *29*, 617–639.
3. Ocali, O., and Atalar, E. (1998). Ultimate intrinsic signal-to-noise ratio in MRI. Magn. Reson. Med. *39*, 462–473.
4. Kraff, O., and Quick, H.H. (2017). 7T: Physics, safety, and potential clinical applications. J. Magn. Reson. Imag. *46*, 1573–1589.
5. Haldar, J.P., Liu, Y., Liao, C., Fan, Q., and Setsompop, K. (2020). Fast submillimeter diffusion MRI using gSlider-SMS and SNR-enhancing joint reconstruction. Magn. Reson. Med. *84*, 762–776.
6. Veraart, J., Fieremans, E., and Novikov, D.S. (2016a). Diffusion MRI noise mapping using random matrix theory. Magn. Reson. Med. *76*, 1582–1593.
7. Manjón, J.V., Coupé, P., Concha, L., Buades, A., Collins, D.L., and Robles, M. (2013). Diffusion weighted image denoising using overcomplete local PCA. PLoS One *8*, e73021.
8. Ma, X., Uğurbil, K., and Wu, X. (2020). Denoise magnitude diffusion magnetic resonance images via variance-stabilizing transformation and optimal singular-value manipulation. Neuroimage *215*, 116852.

CellPress
OPEN ACCESS

9. Cordero-Grande, L., Christiaens, D., Hutter, J., Price, A.N., and Hajnal, J.V. (2019). Complex diffusion-weighted image estimation via matrix recovery under general noise models. Neuroimage *200*, 391–404.

10. Moeller, S., Pisharady, P.K., Ramanna, S., Lenglet, C., Wu, X., Dowdle, L., Yacoub, E., Uğurbil, K., and Akçakaya, M. (2021). Noise reduction with distribution corrected (NORDIC) PCA in dMRI with complex-valued parameter-free locally low-rank processing. Neuroimage *226*, 117539.

11. Veraart, J., Novikov, D.S., Christiaens, D., Ades-Aron, B., Sijbers, J., and Fieremans, E. (2016b). Denoising of diffusion MRI using random matrix theory. Neuroimage *142*, 394–406.

12. Novikov, D., Lemberskiy, G., Baete, S.H., Veraart, J., and Fieremans, E. (2021). System, Method and Computer-Accessible Medium for Facilitating Noise Removal in Magnetic Resonance Imaging. US Patent App. 17/109,050.

13. Jensen, J.H., Helpern, J.A., Ramani, A., Lu, H., and Kaczynski, K. (2005). Diffusional kurtosis imaging: The quantification of non-Gaussian water diffusion by means of magnetic resonance imaging. Magn. Reson. Med. *53*, 1432–1440.

14. Huynh, K.M., Xu, T., Wu, Y., Wang, X., Chen, G., Wu, H., Thung, K.H., Lin, W., Shen, D., and Yap, P.T. (2020). Probing tissue microarchitecture of the baby brain via spherical mean spectrum imaging. IEEE Trans. Med. Imag. *39*, 3607–3618.

15. Zhang, H., Schneider, T., Wheeler-Kingshott, C.A., and Alexander, D.C. (2012). NODDI: practical in vivo neurite orientation dispersion and density imaging of the human brain. Neuroimage *61*, 1000–1016.

16. Huynh, K.M., Xu, T., Wu, Y., Chen, G., Thung, K.-H., Wu, H., Lin, W., Shen, D., and Yap, P.-T.; UNC/UMN Baby Connectome Project Consortium (2019). Probing brain micro-architecture by orientation distribution invariant identification of diffusion compartments. In Medical Image Computing and Computer-Assisted Intervention (MICCA) (Springer), pp. 547–555.

17. Jbabdi, S., Sotiropoulos, S.N., Savio, A.M., Graña, M., and Behrens, T.E.J. (2012). Model-based analysis of multishell diffusion MR data for tractography: How to get over fitting problems. Magn. Reson. Med. *68*, 1846–1855.

18. Jeurissen, B., Leemans, A., Tournier, J.-D., Jones, D.K., and Sijbers, J. (2013). Investigating the prevalence of complex fiber configurations in white matter tissue with diffusion magnetic resonance imaging. Hum. Brain Mapp. *34*, 2747–2766.

19. Tournier, J.-D., Calamante, F., Gadian, D.G., and Connelly, A. (2004). Direct estimation of the fiber orientation density function from diffusion-weighted MRI data using spherical deconvolution. Neuroimage *23*, 1176–1185.

20. Tournier, J.D., Calamante, F., and Connelly, A. (2010). Improved probabilistic streamlines tractography by 2nd order integration over fibre orientation distributions. In International Society for Magnetic Resonance in Medicine (ISMRM).

21. Lemberskiy, G., Baete, S., Veraart, J., Shepherd, T.M., Fieremans, E., and Novikov, D.S. (2019). Achieving sub-mm clinical diffusion MRI resolution by removing noise during reconstruction using random matrix theory. In International Society for Magnetic Resonance in Medicine (ISMRM), *27*.

22. Lemberskiy, G., Baete, S., Veraart, J., Shepherd, T.M., Fieremans, E., and Novikov, D.S. (2020). MRI below the noise floor. In International Society for Magnetic Resonance in Medicine (ISMRM), *28*.

23. Huynh, K.M., Chang, W.-T., Chung, S.H., Chen, Y., Lee, Y., and Yap, P.-T. (2021). Noise mapping and removal in complex-valued multi-channel MRI via optimal shrinkage of singular values. In Medical Image Computing and Computer-Assisted Intervention (MICCAI) (Springer), pp. 191–200.

24. Vannieuwenhoven, N., Vandebril, R., and Meerbergen, K. (2012). A new truncation strategy for the higher-order singular value decomposition. SIAM J. Sci. Comput. *34*, A1027–A1052.

25. Olesen, J.L., Ianus, A., Østergaard, L., Shemesh, N., and Jespersen, S.N. (2023). Tensor denoising of multidimensional MRI data. Magn. Reson. Med. *89*, 1160–1172.

26. Pierre-Antoine, C., Zaineb, A., Alexandre, V., and Philippe, C. (2023). Denoising of fMRI volumes using local low rank methods. In International Symposium on Biomedical Imaging (ISBI), pp. 1–5.

27. Chung, S., Huynh, K., Chen, Y., Yap, P., Goralski, J., Donaldson, S., and Lee, Y. (2022). Ventilation defect percentage comparison between spiral with post-acquisition denoising and Cartesian acquisition for 19F lung magnetic resonance imaging. J. Cyst. Fibros. *21*, S259.

28. Chung, S.H., Huynh, K.M., Goralski, J.L., Chen, Y., Yap, P.-T., Ceppe, A.S., Powell, M.Z., Donaldson, S.H., and Lee, Y.Z. (2023). Feasibility of free-breathing 19F MRI image acquisition to characterize ventilation defects in CF and healthy volunteers at wash-in. Magn. Reson. Med. *90*, 79–89.

29. Dabov, K., Foi, A., Katkovnik, V., and Egiazarian, K. (2007). Image denoising by sparse 3-D transform-domain collaborative filtering. IEEE Trans. Image Process. *16*, 2080–2095.

30. Ramos-Llordén, G., Vegas-Sánchez-Ferrero, G., Liao, C., Westin, C.-F., Setsompop, K., and Rathi, Y. (2021). SNR-enhanced diffusion MRI with structure-preserving low-rank denoising in reproducing kernel Hilbert spaces. Magn. Reson. Med. *86*, 1614–1632.

31. Huynh, K., Wu, Y., Chang, W.-T., and Yap, P.-T. (2024). Optimal Shrinkage Denoising Breaks the Noise Floor in High-Resolution Diffusion MRI (Data at OSF). https://doi.org/10.17605/OSF.IO/F384H.

32. Gavish, M., and Donoho, D.L. (2017). Optimal shrinkage of singular values. IEEE Trans. Inf. Theor. *63*, 2137–2152.

33. Eckart, C., and Young, G. (1936). The approximation of one matrix by another of lower rank. Psychometrika *1*, 211–218.

34. Chatterjee, S. (2015). Matrix estimation by universal singular value thresholding. Ann. Stat. *43*, 177–214.

35. Cai, J.-F., Candès, E.J., and Shen, Z. (2010). A singular value thresholding algorithm for matrix completion. SIAM J. Optim. *20*, 1956–1982.

36. Donoho, D., and Gavish, M. (2014). Minimax risk of matrix denoising by singular value thresholding. Ann. Stat. *42*, 2413–2440.

37. Donoho, D.L. (1995). De-noising by soft-thresholding. IEEE Trans. Inf. Theor. *41*, 613–627.

38. Pruessmann, K.P., Weiger, M., Scheidegger, M.B., and Boesiger, P. (1999). SENSE: sensitivity encoding for fast MRI. Magn. Reson. Med. *42*, 952–962.

39. Eichner, C., Cauley, S.F., Cohen-Adad, J., Möller, H.E., Turner, R., Setsompop, K., and Wald, L.L. (2015). Real diffusion-weighted MRI enabling true signal averaging and increased diffusion contrast. Neuroimage *122*, 373–384.

40. Pizzolato, M., Fick, R., Boutelier, T., and Deriche, R. (2016). Noise floor removal via phase correction of complex diffusion-weighted images: Influence on DTI and q-space metrics. In Medical Image Computing and Computer-Assisted Intervention (MICCAI) (Springer), pp. 21–34.

41. Sprenger, T., Sperl, J.I., Fernandez, B., Haase, A., and Menzel, M.I. (2017). Real valued diffusion-weighted imaging using decorrelated phase filtering. Magn. Reson. Med. *77*, 559–570.

42. Chang, W.-T., Huynh, K.M., Yap, P.-T., and Lin, W. (2020). Navigator-free submillimeter diffusion imaging using multishot-encoded simultaneous multi-slice (MUSIUM). Preprint at arXiv. https://doi.org/10.48550/arXiv.2012.00664.

43. Dietrich, O., Raya, J.G., Reeder, S.B., Ingrisch, M., Reiser, M.F., and Schoenberg, S.O. (2008). Influence of multichannel combination, parallel imaging and other reconstruction techniques on MRI noise characteristics. Magn. Reson. Imaging *26*, 754–762.

44. Griswold, M.A., Jakob, P.M., Heidemann, R.M., Nittka, M., Jellus, V., Wang, J., Kiefer, B., and Haase, A. (2002). Generalized autocalibrating partially parallel acquisitions (GRAPPA). Magn. Reson. Med. *47*, 1202–1210.

45. Garyfallidis, E., Brett, M., Amirbekian, B., Rokem, A., van der Walt, S., Descoteaux, M., and Nimmo-Smith, I.; Dipy Contributors (2014). Dipy, a library for the analysis of diffusion MRI data. Front. Neuroinf. *8*, 8–17.

46. Jeurissen, B., Tournier, J.-D., Dhollander, T., Connelly, A., and Sijbers, J. (2014). Multi-tissue constrained spherical deconvolution for improved analysis of multi-shell diffusion MRI data. Neuroimage *103*, 411–426.

47. Andersson, J.L.R., Skare, S., and Ashburner, J. (2003). How to correct susceptibility distortions in spin-echo echo-planar images: application to diffusion tensor imaging. Neuroimage *20*, 870–888.

48. Caruyer, E., Daducci, A., Descoteaux, M., Houde, J.-C., Thiran, J.-P., and Verma, R.P. (2014). a flexible software library to simulate diffusion MR phantoms. In International Society for Magnetic Resonance in Medicine (ISMRM).

49. Foi, A. (2011). Noise estimation and removal in MR imaging: The variance-stabilization approach. In IEEE International Symposium on Biomedical Imaging (ISBI) (IEEE), pp. 1809–1814.

50. Tournier, J.-D., Calamante, F., and Connelly, A. (2012). MRtrix: diffusion tractography in crossing fiber regions. Int. J. Imag. Syst. Technol. *22*, 53–66.

51. Côté, M.A., Girard, G., Boré, A., Garyfallidis, E., Houde, J.-C., and Descoteaux, M. (2013). Tractometer: towards validation of tractography pipelines. Med. Image Anal. *17*, 844–857.

52. Aydogan, D.B., and Shi, Y. (2021). Parallel transport tractography. IEEE Trans. Med. Imag. *40*, 635–647.

Patterns, Volume *5*

Supplemental information

Optimal shrinkage denoising breaks the noise floor in high-resolution diffusion MRI

Khoi Huynh, Wei-Tang Chang, Ye Wu, and Pew-Thian Yap

Supplementary Materials

Fig. S1. **Data simulation.** Phantom (a) used to simulate noise-free ground truth images (b). The white arrows mark CSF-like regions with isotropic diffusion. Background phase (c), spatially-varying noise map (d), and exemplar channel-sensitivity maps (e, only 4 shown here) used with Eq. (23) to synthesize a realistic dataset.

Fig. S2. **Free-water diffusion.** Normalized free-water diffusion signal from ground truth, noisy, and denoised data with 26 volumes. Shaded regions indicate the original noise floor. The zoomed-in plots highlight the differences between MCC methods.

Fig. S3. **Free-water diffusion.** Normalized free-water diffusion signal from ground truth, noisy, and denoised data with 47 volumes. Shaded regions indicate the original noise floor. The zoomed-in plots highlight the differences between MCC methods.

Fig. S4. **Free-water diffusion.** Normalized free-water diffusion signal from ground truth, noisy, and denoised data with 89 volumes. Shaded regions indicate the original noise floor. The zoomed-in plots highlight the differences between MCC methods.

Fig. S5. **Noise mapping.** Noise map estimated from different approaches. The approach used in our framework (right most) is closest to the ground truth (GT).

Fig. S6. **Background phase estimation.** Example background phases estimated during phase unwinding in different MCC denoising strategies. Also shown are the background phases for noisy data and the ground truth (for simulated data only).

Fig. S7. **MRtrix vs Dipy MP-PCA.** The absolute relative difference between MRtrix *dwidenoise* and Dipy *dipy_denoise_mppca* is less than 1%.

Table S1. **Tractometer statistics for synthetic data.**

Table S2. **Summary of methods evaluated.**

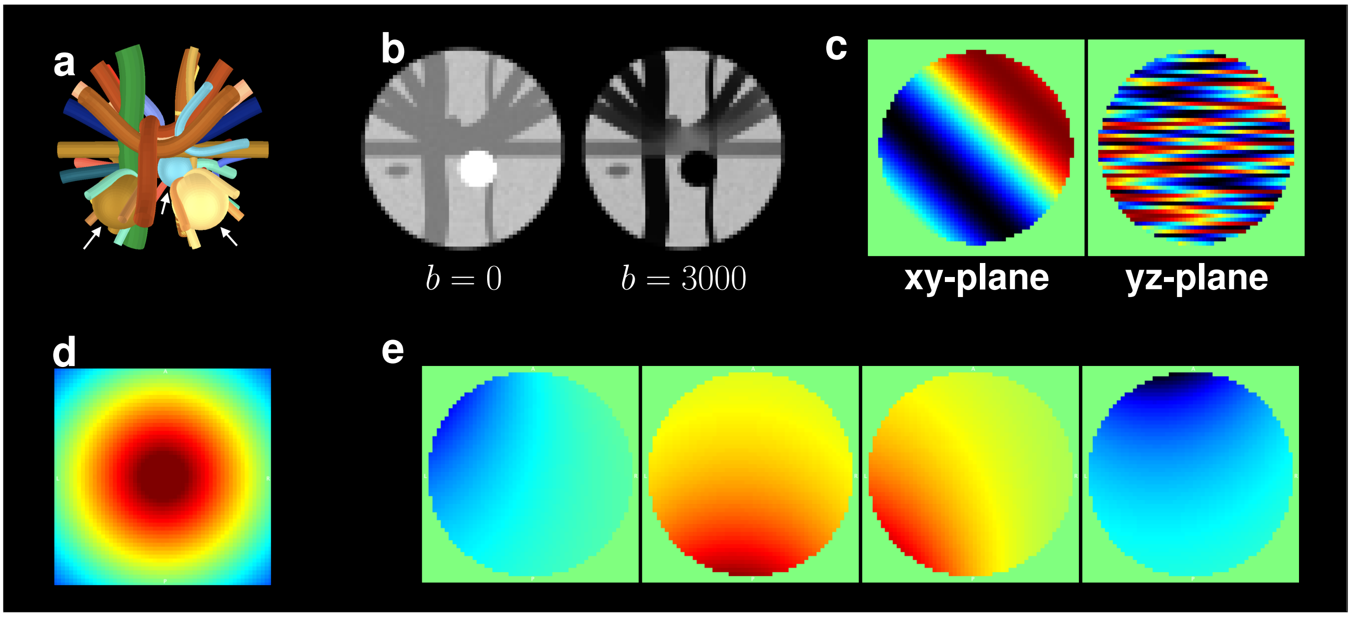

Figure S1: **Data simulation.** Phantom (a) used to simulate noise-free ground truth images (b). The white arrows mark CSF-like regions with isotropic diffusion. Background phase (c), spatially-varying noise map (d), and exemplar channel-sensitivity maps (e, only 4 shown here) used with Eq. (23) to synthesize a realistic dataset.

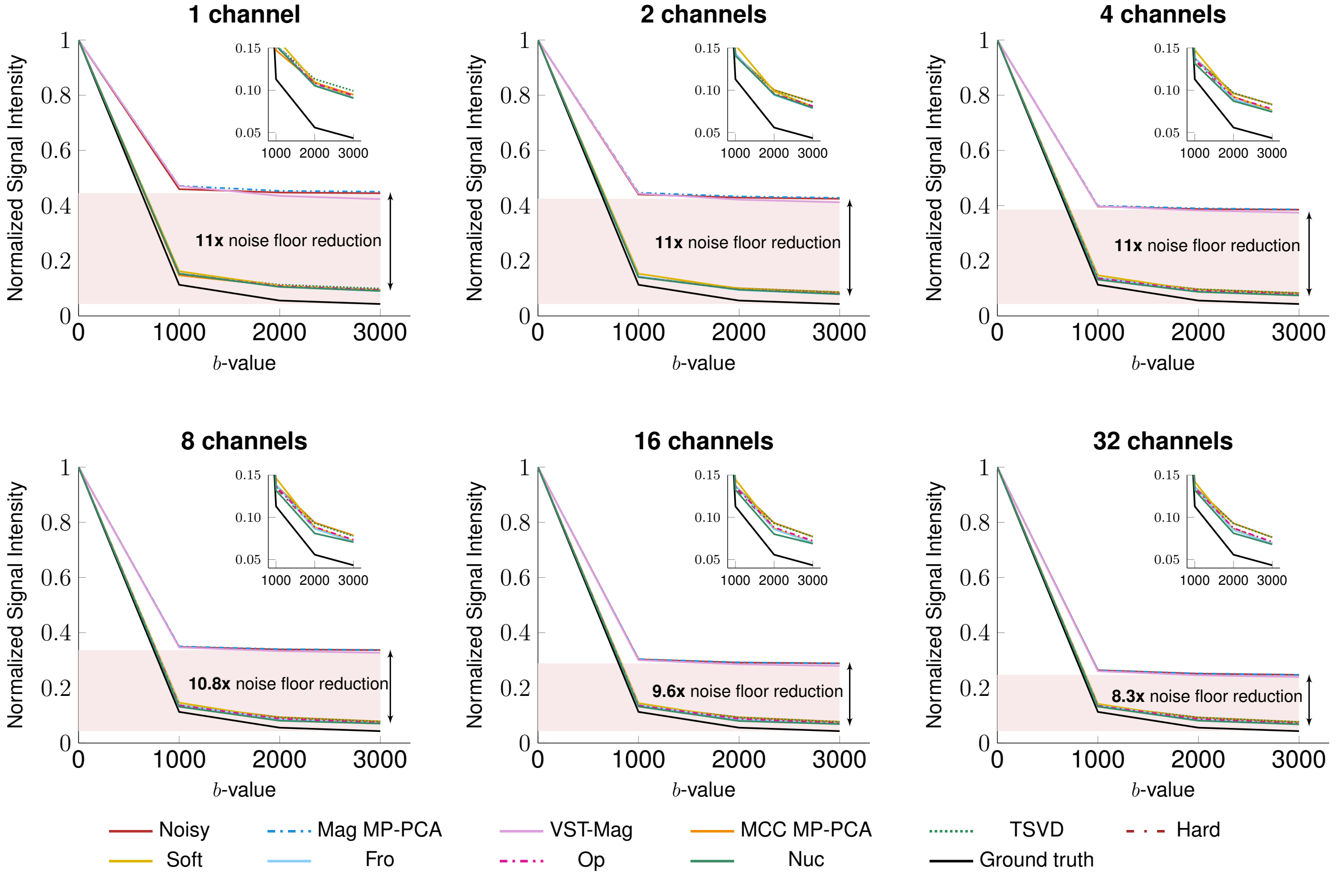

Figure S2: **Free-water diffusion.** Normalized free-water diffusion signal from ground truth, noisy, and denoised data with 26 volumes. Shaded regions indicate the original noise floor. The zoomed-in plots highlight the differences between MCC methods.

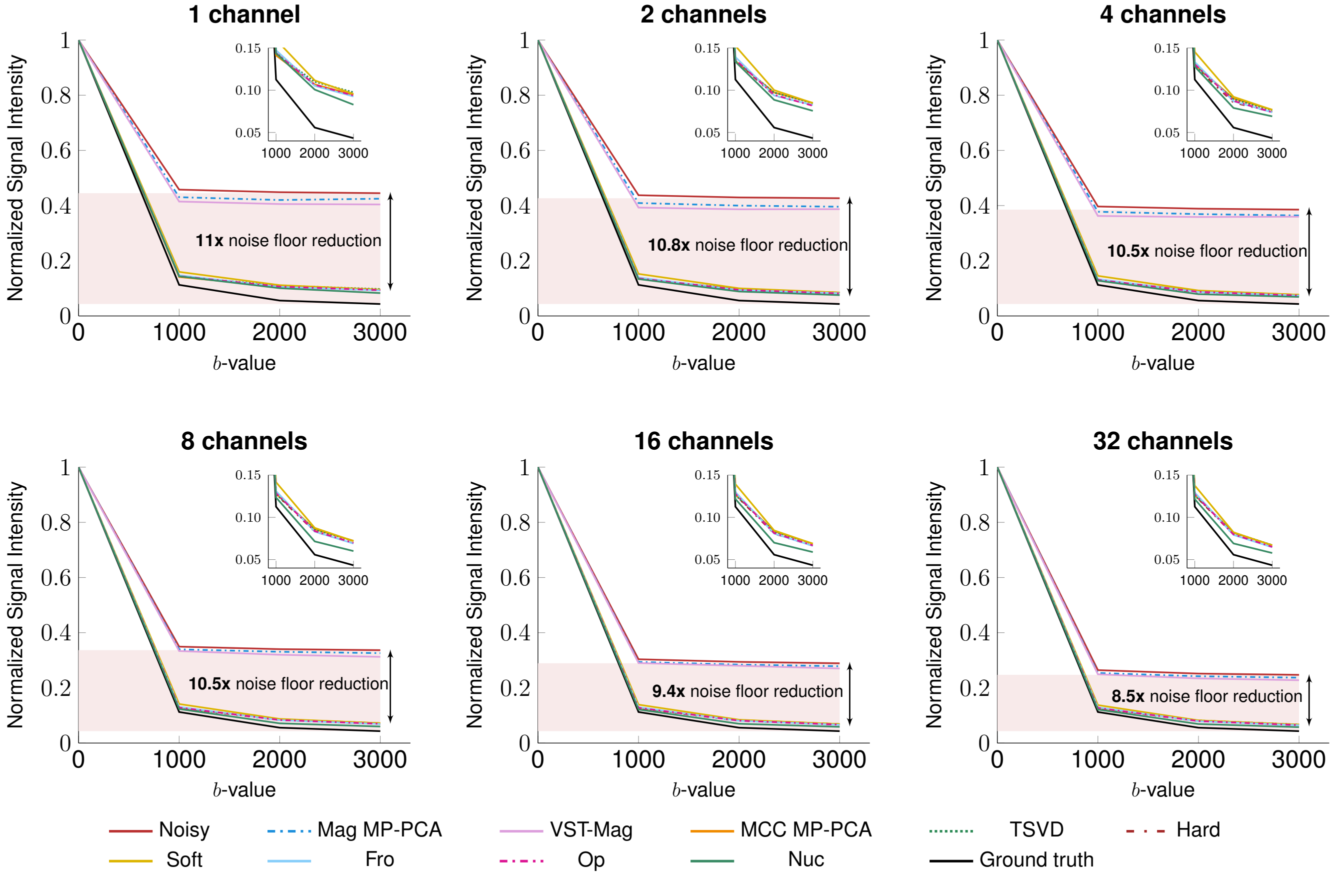

Figure S3: **Free-water diffusion.** Normalized free-water diffusion signal from ground truth, noisy, and denoised data with 47 volumes. Shaded regions indicate the original noise floor. The zoomed-in plots highlight the differences between MCC methods.

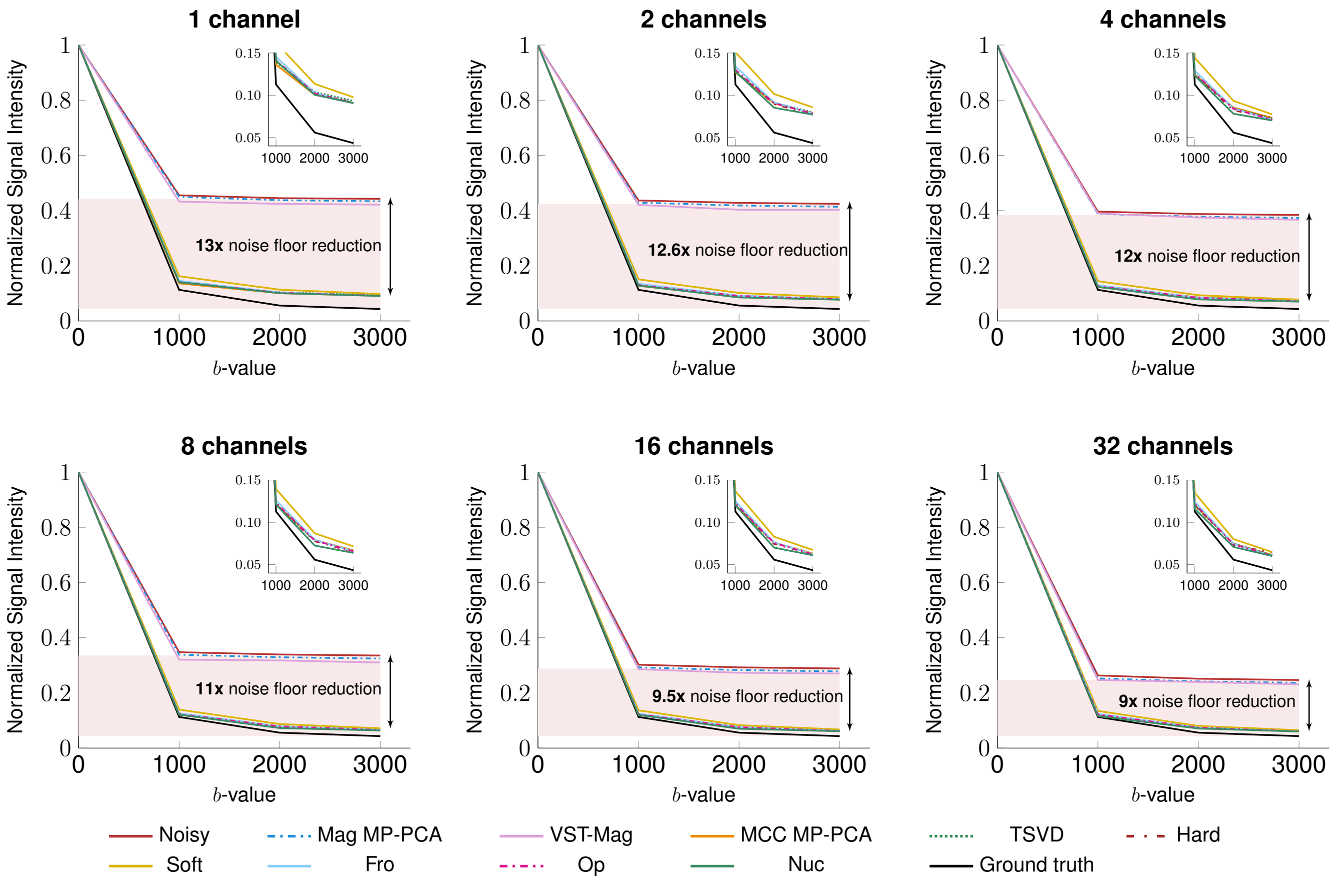

Figure S4: **Free-water diffusion.** Normalized free-water diffusion signal from ground truth, noisy, and denoised data with 89 volumes. Shaded regions indicate the original noise floor. The zoomed-in plots highlight the differences between MCC methods.

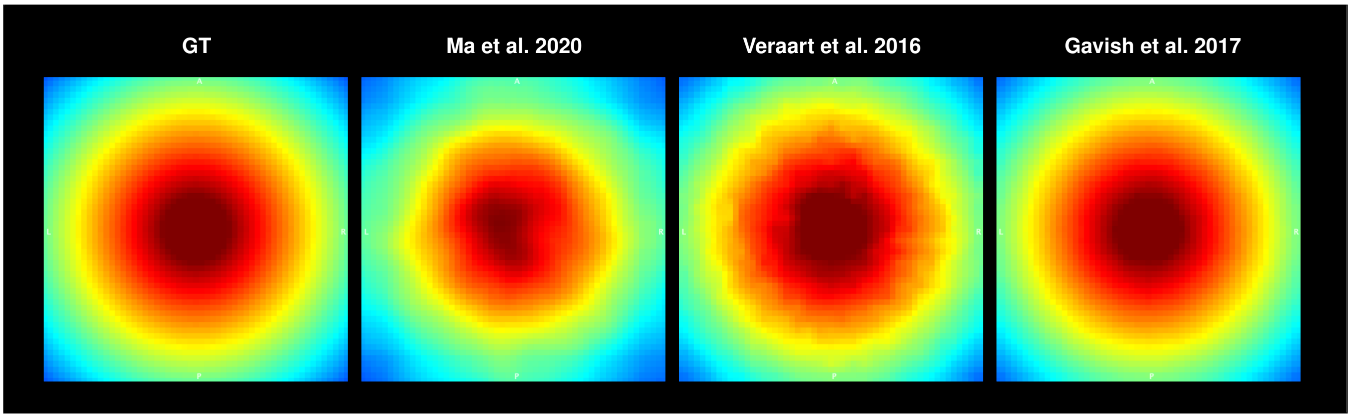

Figure S5: **Noise mapping.** Noise map estimated from different approaches. The approach used in our framework (right most) is closest to the ground truth (GT).

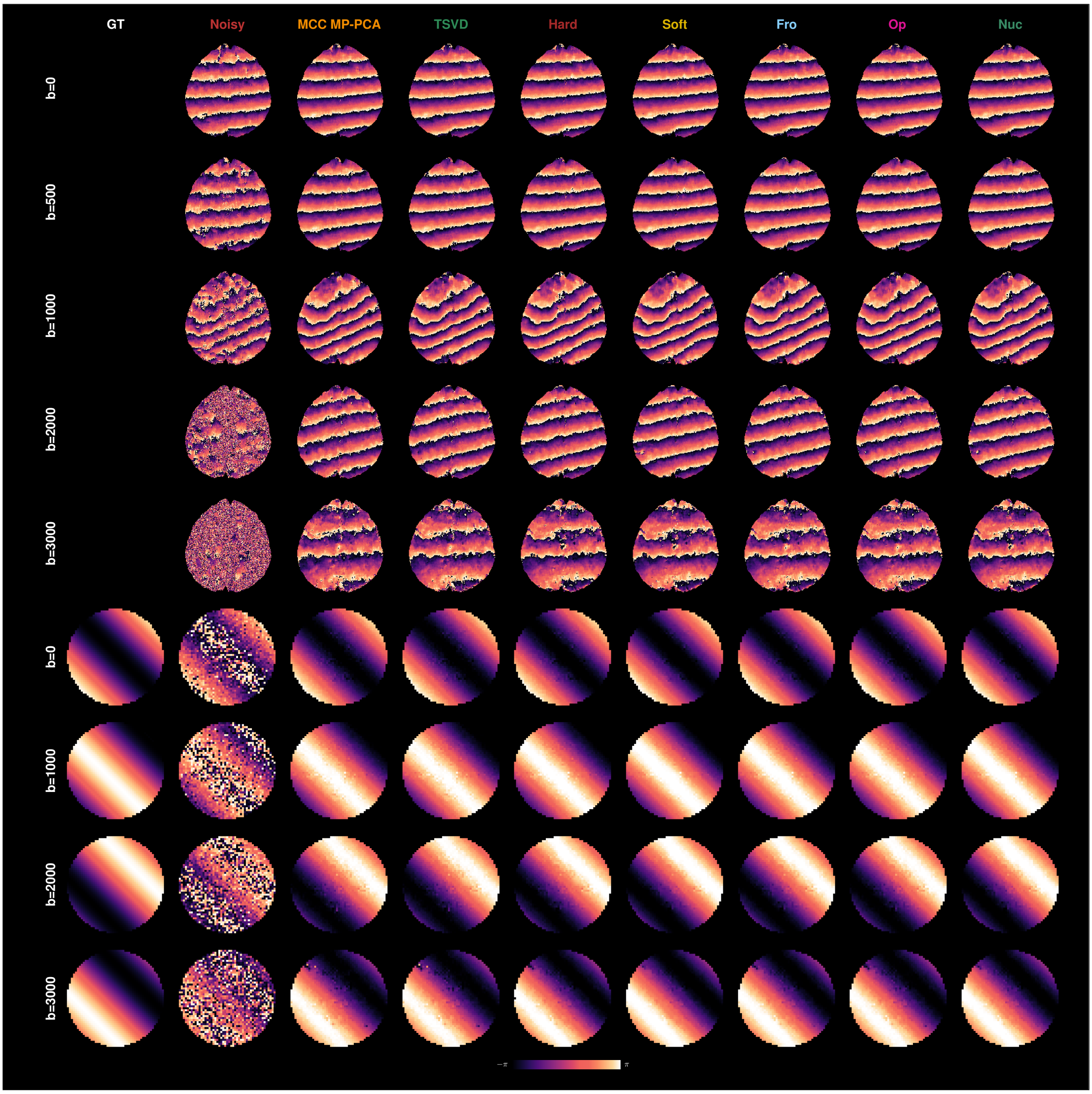

Figure S6: **Background phase estimation.** Example background phases estimated during phase unwinding in different MCC denoising strategies. Also shown are the background phases for noisy data and the ground truth (for simulated data only).

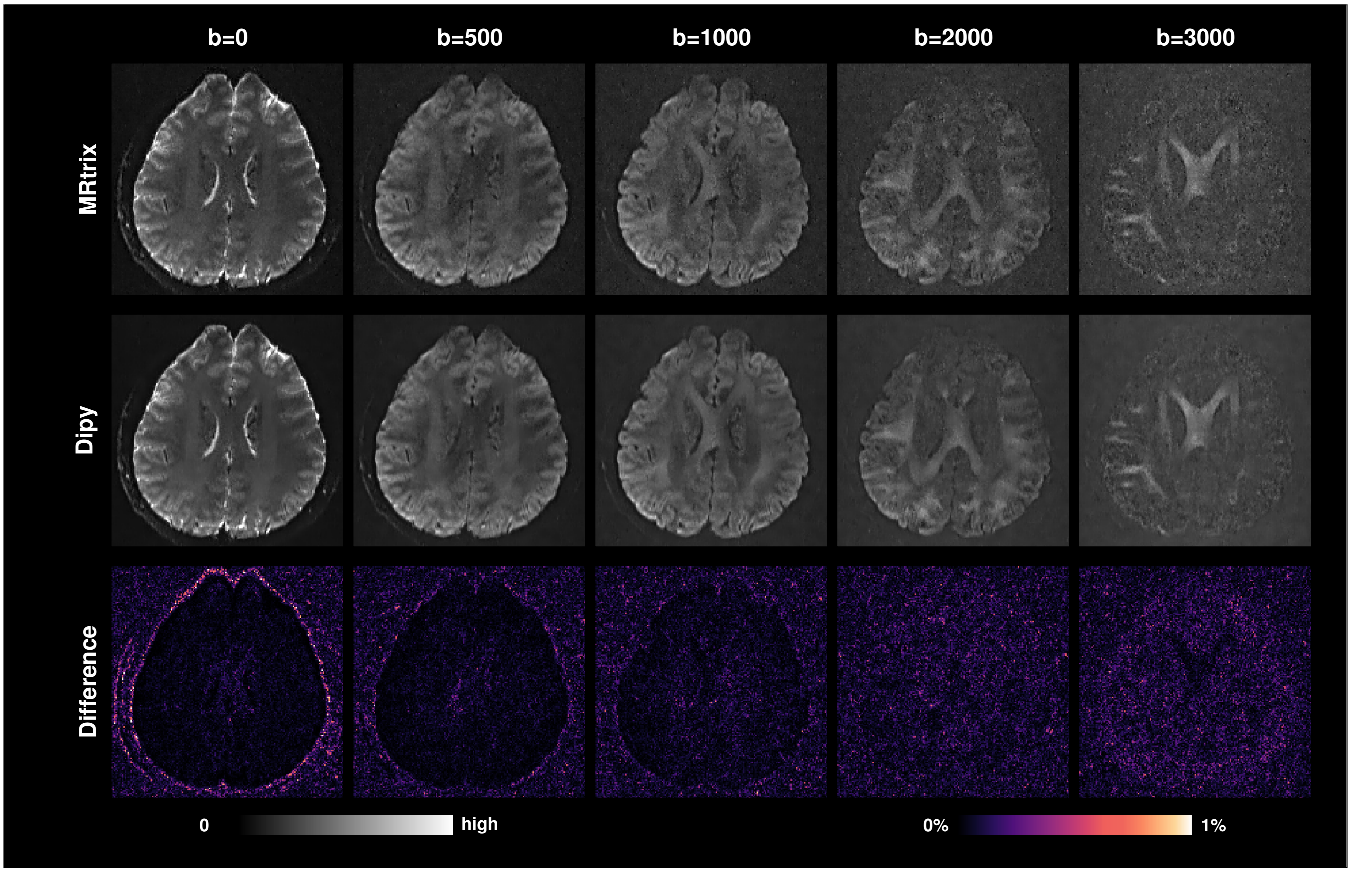

Figure S7: **MRtrix vs Dipy MP-PCA.** The absolute relative difference between MRtrix *dwidenoise* and Dipy *dipy_denoise_mppca* is less than 1%.

Table S1: Tractometer statistics for synthetic data.

	VC(%)	IC(%)	NC(%)	Score	VB	IB
**Ground Truth**	41.00	25.64	33.37	0.48	26	1
**Noisy**	2.17	5.01	92.82	0.04	17	2
**Mag MP-PCA**	16.03	10.13	73.85	0.21	16	2
**VST-Mag**	20.19	14.15	65.66	0.26	15	1
**MCC MP-PCA**	36.75	18.94	44.31	0.43	22	1
**TSVD**	39.58	19.63	40.79	0.46	23	1
**Hard**	37.96	18.07	43.96	0.44	21	1
**Soft**	35.57	19.80	44.63	0.43	21	1
**Fro**	38.03	19.15	42.82	0.45	23	1
**Op**	39.45	19.32	41.23	0.46	24	1
**Nuc**	39.83	19.67	40.50	0.47	25	1

Table S2: Summary of methods evaluated.

	Value	Channel	Channel Decorrelation	Phase Unwinding	Recovery Shrinkage	Noise Estimation
**Mag MP-PCA**[11]	Magnitude	Single	N/A	N/A	Eq. (4)	Eq. (15)
**VST-Mag**[8]	Magnitude	Single	N/A	N/A	Eq. (7)	Two steps, see[8]
**MCC MP-PCA**	Complex	Multi	Yes	Yes	Eq. (4)	Eq. (15)
**TSVD**	Complex	Multi	Yes	Yes	Eq. (11)	Eq. (16)
**Hard**	Complex	Multi	Yes	Yes	Eq. (12)	Eq. (16)
**Soft**	Complex	Multi	Yes	Yes	Eq. (14)	Eq. (16)
**Fro**	Complex	Multi	Yes	Yes	Eq. (7)	Eq. (16)
**Op**	Complex	Multi	Yes	Yes	Eq. (9)	Eq. (16)
**Nuc**	Complex	Multi	Yes	Yes	Eq. (8)	Eq. (16)
